# A New Many-Objective Optimization Approach to Association Rule Mining: The NSGA-II/DE-ARM Algorithm

**DOI:** 10.3390/biomimetics11060362

**Published:** 2026-05-22

**Authors:** Zulfukar Aytac Kisman, Gokhan Demir, Hande Yuksel, Bilal Alatas

**Affiliations:** 1 Technology and Information Management, Firat University, 23119 Elazig, Turkey; 2 Department of Software Engineering, Firat University, 23119 Elazig, Turkey; 222137201@firat.edu.tr (G.D.); hande.yuksel@firat.edu.tr (H.Y.); balatas@firat.edu.tr (B.A.)

**Keywords:** association analysis, bio-based optimization, many-objective evolutionary algorithm, defense industry case

## Abstract

Association rule mining is a fundamental data mining technique for uncovering latent relationships among variables in large-scale datasets. However, conventional approaches rely on single-metric filtering strategies, which are insufficient for capturing the inherent multi-criteria nature of rule quality. To address this limitation, this study formulates ARM as a many-objective optimization problem and proposes a hybrid algorithm, NSGA-II/DE-ARM, that simultaneously optimizes four rule-quality measures: support, confidence, lift, and NetConf. The proposed algorithm enhances the NSGA-II framework by integrating binary differential evolution operators, an adaptive operator selection mechanism, lift-weighted tournament selection, and a constraint-domination principle combined with a dynamic minimum support threshold. Its performance was evaluated using two datasets: a SIPRI–World Bank panel dataset consisting of defense industry and macroeconomic indicators covering 46 items over the 2002–2023 period, and the UCI Mushroom benchmark dataset consisting of 118 items. Across 30 independent runs on the SIPRI–World Bank dataset, NSGA-II/DE-ARM outperformed the Apriori baseline in all four metrics (mean lift = 4.748, confidence = 0.853, support = 0.146, NetConf = 0.789), with large effect sizes (Cohen’s d = 1.77–5.77, *p* < 0.001 in each case). On the Mushroom benchmark dataset, the proposed method also achieved substantial improvements, with Cohen’s d values ranging from 0.93 to 6.16. NSGA-II/DE-ARM generated 68 Pareto-optimal rules in a representative run and achieved the highest hypervolume values on both datasets, with HV = 3.231 for SIPRI–World Bank and HV = 6.262 for Mushroom. These results suggest that NSGA-II/DE-ARM offers decision-makers a broader and more balanced multi-criteria solution set than single-metric filtering approaches.

## 1. Introduction

Data mining is a collection of knowledge discovery techniques that involves extracting highly accurate, potentially useful, understandable, and comprehensible information from large datasets. One of the important research problems in this field is association rule mining (ARM), which aims to reveal multi-attribute correlations in databases. ARM techniques have been successfully applied in various fields such as the healthcare sector, market basket analysis, and recommender systems [1].

Since traditional ARM algorithms generate a large number of rules that are difficult to handle, ARM still remains an open problem [2]. Traditional ARM algorithms, particularly Apriori [3] and Frequent Pattern Growth (FP-Growth), generate frequent itemsets based on minimum support and minimum confidence thresholds. These approaches have two main limitations: (i) the determination of parameters by the user reduces objectivity and requires a trial-and-error process; (ii) performing preliminary filtering based only on a single or limited number of quality measures causes potentially interesting rules to be overlooked [4]. In addition, the fact that these algorithms perform a large number of database scans on large datasets increases the computational cost [5].

On the other hand, the quality of an association rule cannot be adequately expressed by a single measure such as support or confidence alone; many different interestingness measures, such as lift, NetConf, and leverage, also play a decisive role in evaluating the statistical significance of the rule [6]. For this reason, ARM can be formulated as a multi-objective optimization problem in which multiple quality criteria are optimized simultaneously. Multi-objective optimization addresses conflicting objective functions simultaneously.

Evolutionary approaches based on the concept of Pareto optimality provide decision-makers with a solution front rather than a single solution [7]. In particular, methods such as the Non-dominated Sorting Genetic Algorithm II (NSGA-II) [8], the Multi-Objective Evolutionary Algorithm based on Decomposition (MOEA/D) [9], and Strength Pareto Evolutionary Algorithm 2 (SPEA2) [10] provide a suitable framework for the ARM problem thanks to their ability to approximately obtain the Pareto solution set in a single run.

In the evolutionary ARM literature, genetic algorithms, particle swarm optimization, differential evolution, and various metaheuristic approaches have been used. These biologically inspired methods, comprehensively reviewed by Jakšić et al. [11], draw inspiration from evolutionary mechanisms such as natural selection, crossover, mutation, and differential reproduction. NSGA-II mimics the multi-criteria decision-making structure of natural selection through non-dominated sorting, while differential evolution reflects the variation dynamics in population genetics through its mutation mechanism based on vector differences. Nevertheless, the balance between convergence accuracy and solution diversity is still one of the fundamental research issues.

In this context, various approaches have been proposed in the literature. Yuan et al. [12] proposed the multi-objective differential evolution algorithm MODE-FDGM, which integrates a directional generation mechanism to address the problems of low convergence accuracy and weak diversity. Minaei-Bidgoli et al. [13] developed a multi-objective genetic algorithm approach that simultaneously optimizes three criteria—confidence, comprehensibility, and interestingness—for numerical data. Aljehani and Alotaibi [14] introduced the Multi-Threshold Particle Swarm Optimization Association Rule Mining (MPSO4ARM) model, which integrates the Apriori and Particle Swarm Optimization (PSO) algorithms while preserving sensitive rules. Luna et al. [15] addressed the ARM problem from a multi-objective perspective with grammar-guided genetic programming (G3P) models that enable the extraction of numerical and nominal association rules in a single step. In terms of differential evolution-based approaches, Krishna and Ravi [16] proposed the top non-redundant high utility association rule mining (TNR-HUARM) algorithms based on Binary Differential Evolution (BDE) and Adaptive Binary Differential Evolution (ABDE). Ye et al. [17] applied an adaptive parameter strategy based on an improved Whale Optimization Algorithm (WOA) and a Lévy flight mechanism (LWOA) to extract association rules. Berteloot et al. [18], on the other hand, introduced the Cambrian Explosion Algorithm (CEA), which has a large-scale random exploration phase inspired by the evolutionary epoch, to solve the ARM problem. In a more recent study, Sinisterra-Sierra et al. [19] performed causal association rule mining on COVID-19 data using an NSGA-II-based multi-objective evolutionary algorithm.

More recent multi-objective ARM contributions further extend the objective space and the family of metaheuristics employed. Gagnani [20] proposed a soft-computing-based multi-objective ARM framework that simultaneously optimizes multiple interestingness measures on medical datasets. Zheng et al. [21] proposed MOOFARM, a multi-objective optimization-based fuzzy association rule mining approach that aims to improve both quantity-related and quality-related rule metrics in quantitative data by optimizing fuzzy partitioning points. Hu et al. [22] introduced C-MONOA, a cubic-chaotic-map-based multi-objective Nutcracker Optimization Algorithm for mining association rules from mixed continuous and discrete data, using support, confidence, the Kulczynski metric, and comprehensibility as optimization objectives. These works demonstrate that extending the objective space beyond the conventional support-confidence pair is a productive research direction. However, the joint optimization of support, confidence, lift, and NetConf has received limited attention in the existing evolutionary ARM literature. Moreover, the architectural integration of constraint-domination with dynamic support thresholds, lift-weighted tournament selection, and credit-assignment-based adaptive operator selection within a single binary-encoded evolutionary loop distinguishes the present work from these existing approaches.

As noted in comprehensive reviews of multi-objective evolutionary algorithms [7,10], the majority of existing evolutionary ARM studies employ two- or three-objective formulations, typically combining support, confidence, and either lift or comprehensibility. Although recent works have begun to explore four-objective formulations—Hu et al. [22] optimize support, confidence, Kulczynski, and comprehensibility, while Gu et al. [23] include NetConf alongside support—the specific four-objective combination of support, confidence, lift, and NetConf has, to the best of the authors’ knowledge, received limited attention within a single evolutionary loop. In particular, NetConf captures the direction and strength of inter-rule dependency by normalizing the effect of antecedent frequency; its use as an optimization objective, rather than a post hoc filter, enables the evolutionary search to explicitly promote directionally meaningful rules. Furthermore, the combined integration of (i) a constraint domination principle with a dynamic minimum support threshold, (ii) lift-weighted tournament selection, (iii) an external Pareto archive, and (iv) a credit-assignment-based adaptive operator selection mechanism within a single ARM framework has, to the best of the authors’ knowledge, rarely been examined as an integrated design.

Defense expenditures constitute an important component of public fiscal spending and exhibit a multifactorial structure shaped by political, economic, and strategic determinants [24,25]. The existing literature on the determinants of defense spending relies predominantly on traditional econometric techniques such as panel regression, cointegration, and convergence analysis [26], which are primarily designed to estimate average marginal effects under prespecified functional forms. Such approaches are less suited to uncovering non-linear, multi-directional, and conditional co-occurrence patterns among governance, macroeconomic, and military expenditure indicators. To the best of the authors’ knowledge, the application of a many-objective evolutionary ARM framework to defense industry economics remains largely unexplored.

Rationale for adopting a bio-inspired many-objective framework. The choice of a bio-inspired many-objective evolutionary algorithm for closing the two gaps identified above is motivated by four complementary considerations. First, the four-objective ARM problem possesses a discrete, high-dimensional, and non-convex search space (each candidate rule is encoded as a binary vector over the item set, and the objectives—support, confidence, lift, and NetConf—are non-differentiable functions of this encoding); under such conditions, gradient-based and exact methods are not directly applicable, whereas population-based bio-inspired metaheuristics are known to perform competitively [11]. Second, the four objectives are mutually conflicting—maximizing support typically lowers lift, while maximizing confidence may suppress NetConf—so a single optimal rule does not exist. Pareto-based evolutionary algorithms address this by approximating the entire Pareto front in a single run [7,8], thereby providing decision-makers with a diverse set of trade-off solutions rather than a forced scalar compromise. Third, NSGA-II’s non-dominated sorting and crowding-distance mechanisms provide a well-established backbone for diversity preservation in many-objective spaces, while binary differential evolution operators have been shown to be particularly effective in discrete ARM-type search spaces [16]; combining these two complementary mechanisms within a single hybrid loop is therefore a natural design choice. Fourth, in the context of defense industry economics, the goal is not to identify a single “best” rule but to expose a wide spectrum of conditional co-occurrence patterns to decision-makers operating under multi-criteria policy constraints (fiscal, strategic, governance-related); a Pareto-front output is structurally aligned with this decision-support requirement, whereas single-objective or scalarized formulations would discard precisely the diversity that policy analysts need.

Motivated by these gaps, this study proposes NSGA-II/DE-ARM, a bio-inspired many-objective optimization framework for association rule mining that simultaneously optimizes support, confidence, lift, and NetConf within a single binary-encoded evolutionary loop.

The main contributions of this study are summarized in three key points.

1. Methodological contribution: a four-objective ARM formulation. This study formulates association rule mining as a many-objective optimization problem that simultaneously optimizes support, confidence, lift, and NetConf. Whereas the majority of evolutionary ARM studies remain confined to two- or three-objective formulations [7,10], the joint optimization of NetConf together with confidence and lift—rather than its post hoc use as a filtering criterion—has rarely been considered.
2. Algorithmic contribution: a binary NSGA-II/DE hybrid integrating an ARM-specific adaptive operator selection mechanism. The proposed NSGA-II/DE-ARM algorithm couples NSGA-II’s many-objective-capable selection backbone (non-dominated sorting and crowding distance) with binary differential evolution operators that are well-suited to discrete rule-encoding spaces [16]. A credit-assignment-based adaptive operator selection (AOS) mechanism is designed for binary ARM search: the relative effectiveness of crossover and binary DE operators is not known a priori and may shift as the population evolves, so the AOS mechanism updates operator selection probabilities generation-by-generation based on the empirical survival rate of each operator’s offspring. AOS has been previously studied in continuous multi-objective optimization, but its adaptation to the binary ARM setting has received limited attention. The hybrid loop further integrates three supporting mechanisms to improve feasibility handling, selection pressure, and elitist preservation: a constraint-domination principle coupled with a dynamic minimum support threshold, a lift-weighted tournament selection strategy, and an external Pareto archive.
3. Application contribution: many-objective evolutionary ARM analysis of defense industry economics. The proposed framework was applied to a panel dataset compiled from SIPRI, the World Bank, and the Economist Intelligence Unit covering the 2002–2023 period, with the aim of analyzing the macroeconomic and governance-related determinants of defense spending. To the best of the authors’ knowledge, the use of a many-objective evolutionary ARM framework in this domain has received limited attention. The conditional, multi-directional co-occurrence patterns produced by the framework constitute a decision-support output that complements, rather than duplicates, the marginal-effect estimates produced by traditional econometric techniques.

The rest of the study is organized as follows: Section 2 presents the proposed NSGA-II/DE-ARM algorithm. Experimental results are presented in Section 3, the discussion is provided in Section 4, and the overall conclusions are summarized in Section 5.

## 2. Materials and Methods

In this section, the design of the NSGA-II/DE-ARM algorithm, which formulates association rule mining as a four-objective optimization problem, is presented. The proposed method is based on the non-dominated sorting and crowding distance mechanisms of NSGA-II. On top of these, five components are integrated: a differential evolution-based hybrid structure adapted to the binary space, a dynamic support threshold integrated with the constraint domination principle, lift-weighted tournament selection, an external Pareto archive, and credit assignment-based adaptive operator selection.

While each of these components is individually established in the evolutionary optimization literature, their integration within a single ARM framework is motivated by a specific theoretical rationale: each component addresses a distinct challenge arising from the structure of the four-objective binary ARM search landscape, and their combined operation creates a synergy that none achieves in isolation. Specifically: (i) The constraint domination principle with dynamic support thresholds addresses the feasibility challenge—in the binary ARM space, the vast majority of randomly generated candidate rules have zero support or violate minimum frequency requirements; CDP ensures that the evolutionary search prioritizes feasible rules without discarding borderline candidates that may become valuable as the support threshold relaxes over generations. (ii) Binary differential evolution addresses the variation challenge—standard crossover operators in binary spaces tend to produce offspring that are structurally similar to their parents, limiting exploration; DE’s donor-based mutation introduces controlled perturbations that span wider regions of the item-combination space. (iii) Lift-weighted tournament selection addresses the discrimination challenge—in many-objective spaces with four or more objectives, the proportion of mutually non-dominated solutions approaches unity [27], rendering standard crowding-distance tie-breaking ineffective; the lift-weighted mechanism provides a domain-informed secondary criterion that preserves statistically significant rules. (iv) The adaptive operator selection (AOS) mechanism addresses the operator-effectiveness challenge—the relative contribution of crossover and binary DE to offspring survival is problem-dependent and cannot be reliably fixed in advance; AOS dynamically adjusts operator probabilities based on credit assignment from the observed offspring survival rates, eliminating the need for static tuning. (v) The external Pareto archive addresses the elitism challenge—without an unbounded archive, high-quality rules discovered in earlier generations may be lost due to population replacement; the archive ensures monotonic improvement of the approximated Pareto front. The ablation study in Section 3.7 empirically validates this synergy by demonstrating that removing any component degrades overall performance, confirming that the components are not redundant additions but functionally complementary mechanisms.

Notation

Throughout this paper, the following notation is used:

- *T*: total number of transactions in the dataset
- *n*: number of unique items in the dataset
- *r:* a candidate association rule, encoded as a binary vector of length 2n
- *A*(*r*), *C*(*r*): the antecedent and consequent itemsets of rule *r*
- |·|: cardinality (number of items) of an itemset
- *L_max_*: maximum total rule length, i.e., maximum value of |*A*(*r*)| + |*C*(*r*)|
- *LC*: maximum consequent length, i.e., maximum value of |C(r)|
- supp(*r*), conf(*r*), lift(*r*), NetConf(*r*): standard ARM quality measures (defined in Section 2.3)
- nA ∪ C, nA, nC: the number of transactions in which *A*(*r*) ∪ *C*(*r*), *A*(*r*), and *C*(*r*) are observed, respectively
- min_supp(*g*): dynamic minimum support threshold at generation *g* (Section 2.11)
- *sstart*, send: initial and final values of the dynamic support threshold
- *npop*: population size
- *ngen*: total number of generations
- *p_DE_*: probability of selecting the differential evolution operator at each offspring generation step
- *F*: scaling factor of the binary differential evolution operator
- *CR*: crossover rate of the binomial crossover step in binary DE
- *p_mut_*: per-bit mutation probability
- 𝒜: external Pareto archive
- *A_max_*: capacity of the external Pareto archive
- ⊕, ∧, ¬: bitwise XOR, AND, and NOT operators on binary vectors
- ~ Bernoulli(*p*): independent random sampling from a Bernoulli distribution with parameter *p* (i.e., the value 1 is drawn with probability *p* and the value 0 with probability 1 − *p*)
- Var(·): sample variance over a finite set of scalars
- clamp(*x*, *a*, *b*) = min(max(*x*, *a*), *b*): bounding operator
- *U*(0, 1): a random number drawn uniformly from the interval [0, 1]
- *p* ≻ Pareto *q*: Pareto dominance, i.e., fi(*p*) ≥ fi(*q*) for all four objectives, with strict inequality for at least one
- *CV*(*r*): the constraint violation of rule *r* (Section 2.4), used in the constraint domination principle
- ℛ: binary encoding space {0, 1}^2n^
- *R*: the final Pareto-optimal rule set returned by the algorithm

### 2.1. Problem Definition

The ARM problem is formulated as the following constrained many-objective optimization problem:

$${max}_{\;r\;\in \;ℛ}\;\;f(r)\;=\;(f_{1}(r),\;f_{2}(r),\;f_{3}(r),\;f_{4}(r))$$

subject to:

(1) $$g_{1}(r)\;=\;max(0,\;min\_supp(g)\;-\;supp(r))\;\leq \;0\;\left|A\left(r\right)\right|\geq \;1,\;\;\;\;\left|C\left(r\right)\right|\geq \;1\;A\left(r\right)\cap \;C\left(r\right)=\;\emptyset \;\left|A\left(r\right)\right|+\;\left|C\left(r\right)\right|\leq \;L_{max}\;|C(r)|\;\leq \;L_{C}\;$$

where ℛ = {0, 1}^2n^ denotes the binary encoding space (Section 2.2), and the four objectives are:

$f_{1}(r)\;=\;supp(r)$ (support; Equation (3))
$f_{2}(r)\;=\;conf(r)$ (confidence; Equation (3))
$f_{3}(r)\;=\;ln(1\;+\;max(lift(r),\;0))\;-\;ln\;2$ (logarithmic lift)
$f_{4}(r)\;=\;NetConf(r)$ (net confidence; Equation (4))

The transformation in *f*_3_ has two roles: (i) scale compatibility—it narrows the [1, ∞) range of lift to a bounded scale comparable to the other three objectives; (ii) sign normalization—it guarantees that *f*_3_ = 0 when lift(*r*) = 1 (statistical independence between antecedent and consequent).

Constraint (1b) is treated as a soft constraint via the constraint domination principle (Section 2.4); the magnitude of its violation, CV(*r*) = *g*_1_(*r*), serves as the constraint-violation measure used in dominance comparison. The dynamic schedule of min_supp(*g*) is defined in Section 2.11.

Constraints (1c)–(1f) are strictly enforced by the repair operator (Section 2.8) on the binary encoding, so every individual that proceeds to evaluation satisfies them by construction.

Following the convention established by Ishibuchi et al. [28] and consistently adopted in the subsequent evolutionary multi-criterion optimization literature [29], optimization problems with four or more conflicting objectives are referred to as many-objective optimization problems (MaOPs), as opposed to multi-objective problems with two or three objectives. This distinction is operationally meaningful: the proportion of mutually non-dominated solutions in a randomly sampled population approaches one as the number of objectives exceeds three, which sharply reduces the discriminative power of classical Pareto dominance and motivates the use of auxiliary mechanisms such as decomposition, indicator-based selection, or domain-specific tie-breaking rules. Accordingly, the four-objective ARM formulation adopted in this study (support, confidence, lift, and NetConf) is treated as a many-objective problem; the lift-weighted tournament selection introduced in Section 2.6 is, in particular, a tie-breaker designed to address this loss of Pareto discrimination.

### 2.2. Binary Encoding

In a dataset containing a total of n unique items, each individual (rule) is encoded as a binary vector of length $b\in \{0,1{\}}^{2n}$ (Equation (2)):

(2) $$\begin{array}{cc} b= & \underset{⏟}{[a_{1},a_{2},\ldots ,a_{n}},\;\underset{⏟}{c_{1},c_{2},\ldots ,c_{n}]}\\ & \mathrm{Antecedent}(A)\;\mathrm{Consequent}(C) \end{array}$$

If *a_i_* = 1, the *i*-th item is included in the antecedent of the rule; if *c_i_* = 1, it is included in the consequent. This encoding allows the direct application of standard genetic operators through position-based binary representation.

In the SIPRI [30] dataset, *n* = 46 and each individual is represented by a 92-bit vector, whereas in the Mushroom dataset, *n* = 118 and each individual is represented by a 236-bit vector.

The initial population is seeded using the frequent itemsets (FI) obtained by the Apriori algorithm: half of the population is initialized with rules derived from frequent itemsets, while the other half is generated randomly.

This hybrid strategy provides a more meaningful starting point for evolution compared to fully random initialization, while also preserving diversity.

Concrete example. To illustrate the encoding, suppose the dataset contains *n* = 5 unique items: {GDP_High, GDPPC_High, RL_High, CoC_High, PC_High}. The 10-bit individual $\begin{array}{cc} b= & \underset{⏟}{[0,1,1,0,0}|\;\underset{⏟}{0,0,0,1,0]}\\ & \mathrm{antecedent}\;\mathrm{half}\;consequent\;half \end{array}$ encodes the rule {GDPPC_High, RL_High} → {CoC_High}, since positions 2 and 3 are active in the antecedent half (corresponding to GDPPC_ High and RL_ High) and position 4 is active in the consequent half (corresponding to CoC_ HIGH). The rule has |*A*(*r*)| = 2, |*C*(*r*)| = 1, *A*(*r*) ∩ *C*(*r*) = ∅, and total length |*A*(*r*)| + |*C*(*r*)| = 3, satisfying the structural constraints of Equation (1).

### 2.3. Objective Function Computation

The objective values of each individual are obtained through count-based computation on the dataset. Let *T* be the total number of transactions, $n_{A\cup C}$ the number of transactions in which the antecedent and consequent of the rule are observed together, $n_{A}$ the number of transactions in which the antecedent is observed, and $n_{C}$ the number of transactions in which the consequent is observed; support, confidence, and lift are defined in Equation (3), and NetConf is defined in Equation (4):

(3) $$\mathrm{supp}\left(r\right)=\frac{n_{A\cup C}}{T},\;\mathrm{conf}\left(r\right)=\frac{n_{A\cup C}}{n_{A}},\;\mathrm{lift}(r)=\frac{\mathrm{conf}(r)}{n_{C}/T}$$

(4) $$\mathrm{NetConf}(r)=\frac{\mathrm{supp}(r)-\mathrm{supp}(A)\cdot \mathrm{supp}(C)}{\mathrm{supp}(A)\cdot (1-\mathrm{supp}(A))}$$

NetConf (Net Confidence) measures the true relational significance of a rule more accurately by normalizing the effect of antecedent frequency, which support and confidence cannot ignore.

### 2.4. Fast Non-Dominated Sorting and CDP

Individuals in the population are ranked using fast non-dominated sorting integrated with the CDP proposed by Deb et al. [8]. The dominance relationship between two individuals, *p* and *q*, is defined as follows:

**Definition 1 (CDP-Dominance).**  $p\;{≻\;}_{CDP}q$ *It holds when one of the following conditions is satisfied:*

*1. If p is feasible (CV_p_ = 0) and q is infeasible (CV_q_ > 0)*

*2. If both are infeasible and CV_p_ < CV_q_*

*3. If both are feasible and p ≻ _Pareto_ q (i.e., f_i_(p) ≥ f_i_(q) for all objectives and f_i_(p) > f_i_(q) for at least one objective)*

Using this rule set, the fast non-dominated sorting algorithm with $O(MN^{2})$ complexity is implemented by using the dominates_cdp function in the dominance check (where *M* is the number of objectives and *N* is the population size).

### 2.5. Crowding Distance

To preserve the diversity of individuals on the same Pareto front, the crowding distance is calculated according to Equation (5). Let $F_{k}$ denote the set of individuals on the k-th front, and *m* the number of objectives:

(5) $$d(i)=\sum_{j=1}^{m}\frac{f_{j}^{\left(i+1\right)}-f_{j}^{\left(i-1\right)}}{f_{j}^{max}-f_{j}^{min}}$$

Infinite distance is assigned to boundary individuals (i=0 and $i=|F_{k}|-1)$. In each objective dimension, individuals are sorted, and the normalized distance between neighboring solutions is accumulated. Individuals with high crowding distance represent solutions in sparser regions and are prioritized for preserving diversity.

### 2.6. Lift-Weighted Tournament Selection

In binary tournament selection, the winner between two randomly selected individuals is determined according to the following hierarchical criteria:

1. Front rank: The individual with the lower front number is preferred.
2. Crowding distance: If they are on the same front, the individual with the higher crowding distance is preferred.
3. Lift value (if LB is active): In the case of equal crowding distance, the individual with the higher lift value is preferred.

The third criterion (lift-weighted selection—LB) is a component added to standard NSGA-II. This mechanism promotes the preservation of rules with high relational significance.

### 2.7. Differential Evolution (DE) Hybridization

In addition to the traditional single-point crossover operator of NSGA-II, binary DE operators are applied probabilistically. In each generation, if the condition $U(0,1)<p_{DE}$ (DE probability) is satisfied, the DE operator is applied; otherwise, standard crossover is used.

Binary DE/rand/1/bin Adaptation:

The classical DE formulation in continuous space, $v=x_{r_{1}}+F\cdot (x_{r_{2}}-x_{r_{3}})$, is adapted to binary space as follows:

Base and difference vectors: From three distinct individuals randomly selected from the population, $r_{1},r_{2},r_{3}$:

$$d=x_{r_{2}}⊕x_{r_{3}}(\mathrm{bitwise}\;\mathrm{XOR}\;-\;\mathrm{difference}\;\mathrm{vector})$$

Scaling mask: A Bernoulli mask **M** ∈ {0, 1}^{2n} is generated using the scaling factor *F*. Each entry *M_i* of this mask is sampled independently as *M_i* ~ Bernoulli(*F*) for *i* = 1, …, 2n, meaning that *M_i* takes the value 1 with probability *F* and 0 with probability 1 − *F*.

Donor vector: $v=x_{r_{1}}⊕(M\wedge d)$

Crossover (binomial): Binary trial vector:

$$u_{i}=\{\begin{array}{cc} v_{i} & \mathrm{if}\;U(0,1)<CR\;\mathrm{or}\;i=j_{\mathrm{rand}}\\ x_{i}^{\mathrm{target}} & \mathrm{otherwise} \end{array}$$

where crossover rate (*CR*) is the crossover rate, and $j_{\mathrm{rand}}$ is a random index that guarantees the use of the donor vector in at least one dimension.

The main advantage of this hybrid structure is that the XOR-based difference operator captures the structural differences among the existing solutions in the population and uses this information in generating new solutions. Compared to standard single-point crossover, it exhibits a broader search behavior.

### 2.8. Repair Operator

After each genetic operator, a four-stage repair procedure is applied to ensure the validity of the generated offspring individuals:

Step 1—Overlap cleaning: Bits that are simultaneously active in both the antecedent and the consequent are identified. In case of conflict, the antecedent is prioritized; the consequent bits are set to zero.

overlap=A∧C;C←C∧¬overlap

Step 2—Gap filling: If the antecedent or consequent is completely empty, a random item is activated.

$$\left|A|=0\Rightarrow a_{j}\leftarrow 1\;(\mathrm{random}\;j);|C|=0\Rightarrow c_{k}\leftarrow 1\;(\mathrm{random}\;k\notin A\right)$$

Step 3—Consequent length constraint: If $|C|>L_{C}$, the excess consequent bits are randomly set to zero.

$$\left|C|>L_{C}\Rightarrow C^{\prime }=\mathrm{random}\_\mathrm{selection}(C,L_{C}\right)$$

Step 4—Total length constraint: If $|A|+|C|>L_{max}$, bits are first removed from the antecedent (the consequent is preserved).

$$|A|+|C|>L_{max}\Rightarrow A^{\prime }=\mathrm{random}\_\mathrm{trim}(A,L_{max}-|C|);|A^{\prime }|\geq 1$$

This hierarchical repair guarantees the strict satisfaction of all structural constraints (A∩C=∅, |A|≥1, |C|≥1, $|A|+|C|\leq L_{max}$).

### 2.9. External Pareto Archive

A fixed-capacity external archive ($|A|\leq A_{max}$) is used to preserve elitist solutions across generations. The archive update procedure is as follows:

1. In each generation, feasible (CV = 0) Front-1 solutions are considered as candidates for the archive.
2. For each candidate *x*:
  - If there is a solution in the archive that dominates *x*, the candidate is rejected.
  - If *x* dominates one or more solutions in the archive, the dominated solutions are removed from the archive and *x* is added.
  - Otherwise (non-dominated and not dominating), *x* is added.
3. In case of capacity overflow ($|A|>A_{max}$), the solutions with the lowest lift values are removed.

This mechanism ensures that the best non-dominated solutions found during evolution are not lost. In the final generation, the population and the archive are merged, and a final Pareto filtering is applied to obtain the final rule set.

### 2.10. Stability Measurement (Bootstrap)

The stability of each rule in the dataset is evaluated using the bootstrap resampling method. Let B be the number of repetitions and α be the sampling ratio:

1. *B* subsamples are repeatedly drawn from the dataset (of size α·T, with replacement).
2. In each subsample, the support and confidence values of the rule are recalculated; let sb and cb denote the support and confidence values, respectively, computed on the b-th bootstrap subsample (*b* = 1, …, *B*).
3. The stability score is calculated as the sum of the variances of support and confidence:

$$\mathrm{stability}(r)=\mathrm{Var}(\{s_{b}{\}}_{b=1}^{B})+\mathrm{Var}(\{c_{b}{\}}_{b=1}^{B})$$

A low stability(r) value indicates that the rule shows consistent performance across different subsamples in terms of both support and confidence and therefore has a low risk of overfitting to the data. In this study, B=500 and α=0.6 were used.

### 2.11. Dynamic Minimum Support Threshold

To gradually expand the search space during the evolutionary process, the minimum support threshold is linearly decreased according to the generation number, as in Equation (6):

(6) $$\min\backslash \_\mathrm{supp}(g)=s_{\mathrm{start}}-\frac{g}{n_{\mathrm{gen}}}\times (s_{\mathrm{start}}-s_{\mathrm{end}})$$

In the initial generations, a high support threshold ensures that the population starts with strict and frequent rules. Toward the later generations, lowering the threshold enables the discovery of rarer but high-lift rules. In this study, $s_{\mathrm{start}}=0.08$ and $s_{\mathrm{end}}=0.03$ were used.

### 2.12. Adaptive Operator Selection (AOS)

Binary operators that mimic two fundamental mechanisms of biological evolution—crossover (recombination) and differential mutation—are used in NSGA-II/DE-ARM. While the operator selection probability ($p_{DE}$) is kept fixed in traditional approaches; in this study, a credit assignment-based adaptive operator selection (AOS) mechanism is proposed [31,32].

The AOS mechanism operates as follows:

- Labeling: Each offspring is tagged with the operator that produced it (DE or CX—single-point crossover).
- Credit assignment: After survival selection, the operator labels of the offspring that advance to the next generation are counted.
- Success rate: The success rate of each operator is calculated as the number of surviving offspring divided by the total number of offspring generated.
- Probability update: The DE probability is updated according to Equation (7) using an exponential moving average (EMA):

(7) $$p_{DE}^{\left(g+1\right)}=\mathrm{clamp}(\alpha_{\mathrm{EMA}}\cdot \frac{r_{DE}}{r_{DE}+r_{CX}}+(1-\alpha_{\mathrm{EMA}})\cdot p_{DE}^{\left(g\right)},\;p_{min},\;p_{max})$$

where $\alpha_{\mathrm{EMA}}=0.1$ (smoothing factor), $p_{min}=0.40$, and $p_{max}=0.85$. These parameter values were determined through a grid search in which five different configurations were tested with five seeds each. In Equation (7), *r_DE_* and *r_CX_* denote the empirical survival rates of DE-generated and crossover-generated offspring, respectively, computed in the current generation as the number of surviving offspring divided by the total number of offspring produced by that operator.

This mechanism is motivated by the fact that the relative effectiveness of crossover and binary DE in producing surviving offspring is problem-dependent and may also drift as the population evolves; rather than fixing the operator selection probability a priori, AOS learns it online from the empirical survival rates of each operator’s offspring.

### 2.13. Algorithm Flow

Algorithm 1 presents the pseudocode of NSGA-II/DE-ARM:
**Algorithm 1:** NSGA-II/DE-ARMInput: Transaction set T, parameter set Output: Pareto-optimal rule set R, derived from the final population P and the external archive 𝒜 after the evolutionary loop terminates 1:   FI ← Apriori(T, s_start) 2:   P ← Seed_and_RandomInitialize(FI, n_pop) 3:   𝒜 ← ∅
; p_DE ← p_DE_0_ 4:   for g = 0 to n_gen − 1 do 5:    min_s ← DynamicSupportThreshold (g) 6:    Evaluate(P, min_s) 7:    F ← FastNonDominatedSort_CDP(P) 8:    for k = 1 to n_pop do 9:      p_1_, p_2_ ← Lift-Weighted_TournamentSelection(P, F) 10:       if U(0,1) < p_DE then 11:         child ← BinaryDE_rand_1_bin(p_1_, P, F, CR) 12:       else 13:         child ← Single-Point_Crossover (p_1_, p_2_) 14:       end if 15:       child ← Repair(BitFlip(child, p_mut)) 16:     end for 17:     *P_g_*_+1_ ← NSGA-II_ Survival(P ∪ Q) 18:     p_DE ← AOS Update (Q, *P_g_*_+1_, p_DE) 19:     ArchiveUpdate (𝒜, *P_g_*_+1_, A_max) 20:   end for 21:   Final ← ParetoFiltering(P ∪ 𝒜) 22:   Final.stability ← BootstrapStability(Final, T) 23:   R ← Final 24:   return R

Inputs and parameters used in Algorithm 1.

The transaction set *T* is the only data input. The “parameter set” passed to the algorithm consists of the following pre-configured values, all of which are reported in Table 3: npop, ngen, pmut, pDE0 (initial DE probability), pmin, pmax (lower and upper bounds for *p_DE_* under AOS), α_EMA (AOS smoothing factor), *F* (DE scaling factor), *CR* (binomial crossover rate), *sstart*, send (initial and final values of the dynamic minimum support threshold), *Lmax*, LC (maximum rule length and maximum consequent length), and Amax (archive capacity). The output of the algorithm is the Pareto-optimal rule set *R*, returned in the final line as the result of ParetoFiltering(P ∪ 𝒜) (i.e., *R* is the set assigned to Final and returned).

Note that R is not updated inside the evolutionary loop; the iteratively updated state variables are the working population P and the external Pareto archive 𝒜. R is constructed only once, at the post-loop finalization stage (lines 21–24), by extracting the non-dominated front from P ∪ 𝒜 and annotating each rule with its bootstrap stability score.

The functions invoked in Algorithm 1 are defined as follows:

Apriori(*T*, *sstart*): the classical Apriori algorithm [3] applied to transaction set *T* with minimum support sstart; returns the set *FI* of frequent itemsets.

Seed_and_RandomInitialize(*FI*, *n*_pop_): initializes a population of size npop in which half of the individuals are seeded with rules derived from the frequent itemsets in *FI* and the remaining half are generated by independent uniform sampling over {0, 1}^{2n}, followed by repair (Section 2.8).

DynamicSupportThreshold(g): returns the value of min_supp(g) at generation g according to Equation (6).

Evaluate(P, min_s): computes the four objective values f_1_–f_4_ (Equations (1), (3) and (4)) and the constraint-violation value CV (Section 2.4) for every individual in population P, using min_s as the active minimum-support threshold.

FastNonDominatedSort_CDP(P): partitions population P into Pareto fronts F_1_, F_2_, … using the constraint-dominance relation defined in Section 2.4, and assigns each individual a front rank and a crowding distance (Section 2.5).

Lift-Weighted_TournamentSelection(*P*, *F*): returns two parents drawn by binary tournament from P, applying the hierarchical winner-determination rule (front rank → crowding distance → lift) defined in Section 2.6.

BinaryDE_rand_1_bin(*p*_1_, *P*, *F*, *CR*): produces a single offspring from parent p_1_ using the binary DE/rand/1/bin operator (Section 2.7), where two additional individuals are randomly selected from P to form the difference vector and F is the scaling factor.

Single-Point_Crossover(*p*_1_, *p*_2_): applies standard single-point crossover to two binary parent strings.

BitFlip(*x*, *p_mut_*): flips each bit of *x* independently with probability *p_mut_*.

Repair(*x*): enforces the rule-structure constraints by removing antecedent–consequent overlap, filling empty rule parts, and trimming antecedent or consequent items that exceed the allowed limits.

NSGA-II_Survival(P ∪ Q): elitist (μ + λ) survival selection that merges the current population *P* and the offspring set *Q*, applies fast non-dominated sorting with the constraint-dominance relation, and retains the top npop individuals using crowding distance as the secondary criterion (Section 2.4 and Section 2.5).

AOS_Update(Q, *P_g_*_+1_, *p_DE_*): updates pDE according to Equation (7), where the empirical survival rates *r_DE_* and *r_CX_* are computed as the number of DE-labeled (respectively, CX-labeled) offspring in Q that survive into *P_g_*_+1_, divided by the total number of DE-labeled (respectively, CX-labeled) offspring generated in this generation.

ArchiveUpdate(𝒜, *P_g_*_+1_, *A_max_*): updates the external archive by admitting feasible Front-1 candidates, removing dominated solutions, and pruning low-lift entries when the archive exceeds its capacity.

ParetoFiltering(*P* ∪ 𝒜): merges the final population *P* with the external archive 𝒜 and returns the non-dominated front of the union.

BootstrapStability(*R*, *T*): computes a post hoc stability score for each final rule by estimating the variance of its support and confidence over bootstrap subsamples.

### 2.14. Comparison Algorithms

To evaluate the performance of the proposed NSGA-II/DE-ARM algorithm, two well-known multi-objective evolutionary algorithms were used as comparison methods: MOEA/D and SPEA2. These algorithms were selected because they represent two different selection philosophies in multi-objective optimization. MOEA/D follows a decomposition-based search strategy, whereas SPEA2 follows a strength-based elitist selection strategy. Therefore, their inclusion enables the proposed NSGA-II/DE-ARM framework to be compared against alternative Pareto search mechanisms.

For comparability in the ARM setting, all three evolutionary algorithms used the same binary rule encoding, objective functions, repair operator, dynamic minimum support schedule, bootstrap-based stability evaluation, and external archive capacity. This design isolates the effect of the environmental selection and population update mechanisms while keeping the association rule representation and rule validity constraints identical across algorithms. Therefore, the comparison should be interpreted as a controlled algorithmic comparison under a shared ARM representation rather than as a comparison of fully independent off-the-shelf implementations.

Methodological justification for the shared-infrastructure design. This controlled experimental design follows the established practice in the multi-objective evolutionary algorithm (MOEA) benchmarking literature. As emphasized by Deb et al. [8] and Zhou et al. [7], isolating the selection and survival mechanisms while keeping representation and variation operators constant is the standard methodology for evaluating the relative merit of different MOEA paradigms. Using fully independent, off-the-shelf implementations would confound multiple factors simultaneously—differences in encoding, constraint handling, and variation operators would make it impossible to attribute performance differences to the core algorithmic mechanisms. In contrast, the shared-infrastructure approach adopted here ensures that performance differences are attributable solely to the selection paradigm: NSGA-II’s non-dominated sorting with crowding distance versus MOEA/D’s Tchebycheff decomposition versus SPEA2’s strength-based ranking with density truncation. Critically, the NSGA-II/DE-ARM-specific components—lift-weighted tournament selection (LB) and adaptive operator selection (AOS)—are not shared with the comparison algorithms; their isolated contributions are quantified in the ablation study (Section 3.7). Furthermore, a classical ARM baseline (Apriori with multi-metric filtering) is included in all comparisons (Table 8 and Table 13), providing an independent, non-evolutionary reference point that validates the overall advantage of evolutionary multi-objective approaches.

#### 2.14.1. MOEA/D for Association Rule Mining

The Multi-Objective Evolutionary Algorithm based on Decomposition (MOEA/D) decomposes a multi-objective optimization problem into a set of scalar subproblems. Each subproblem is associated with a weight vector and is optimized by using information from its neighboring subproblems. In the ARM context, each individual represents a candidate association rule encoded as a binary vector. The four rule quality objectives—support, confidence, transformed lift, and NetConf—are evaluated for each candidate rule.

In this study, MOEA/D operates as follows. First, a population of binary-encoded rules is initialized using the same seeding strategy as NSGA-II/DE-ARM. A set of uniformly distributed weight vectors is generated, and for each weight vector, a neighborhood is defined based on Euclidean distance in the weight space. At each generation, offspring rules are generated by applying the binary differential evolution and mutation operators. Each offspring is repaired to satisfy the structural rule constraints and then evaluated using the four objective functions. The offspring is compared with the current solutions of the corresponding neighboring subproblems using a scalar aggregation function. If the offspring improves the scalar fitness value of a neighboring subproblem, the corresponding solution is replaced. After the population update, feasible non-dominated solutions are transferred to the external Pareto archive.

Algorithm 2 summarizes the MOEA/D procedure used in this study.
**Algorithm 2:** MOEA/D for ARMInput: Transaction set T, parameter set, weight vectors W Output: Pareto-optimal rule set R 1: FI ← Apriori(T, s_start) 2: P ← Seed_and_RandomInitialize(FI, n_pop) 3: Initialize weight vectors W and neighborhood structure B 4: 𝒜 ← ∅
5: for g = 0 to n_gen − 1 do 6:   min_s ← DynamicSupportThreshold(g) 7:   Evaluate(P, min_s) 8:   for each subproblem i = 1 to n_pop do 9:     Select parent solutions from neighborhood B(i) 10:      child ← BinaryDE_rand_1_bin(parent, P, F, CR) 11:      child ← Repair(BitFlip(child, p_mut)) 12:      Evaluate(child, min_s) 13:      for each neighboring subproblem j ∈ B(i) do 14:       if child improves the scalar aggregation value of P[j] then 15:         P[j] ← child 16:       end if 17:      end for 18:   end for 19:   ArchiveUpdate(𝒜, P, A_max) 20: end for 21: R ← ParetoFiltering(P ∪ 𝒜) 22: return R

#### 2.14.2. SPEA2 for Association Rule Mining

The Strength Pareto Evolutionary Algorithm 2 (SPEA2) is an elitist multi-objective evolutionary algorithm that ranks candidate solutions using both dominance strength and density information. Unlike MOEA/D, which decomposes the problem into scalar subproblems, SPEA2 maintains an external archive and evaluates each solution according to how many other solutions it dominates and how densely populated its surrounding region is.

In the ARM context, each SPEA2 individual is a binary-encoded association rule. At each generation, the population and archive are combined, and the strength value of each rule is calculated based on the number of rules it dominates. Then, a raw fitness value is assigned by considering the strength values of the solutions that dominate it. To preserve diversity, SPEA2 also computes a density estimate using the distance to the k-th nearest neighbor in the objective space. The final fitness value is obtained by combining raw fitness and density information. Feasible non-dominated rules are prioritized, and if the archive exceeds its maximum capacity, a truncation procedure removes solutions from crowded regions of the objective space.

Algorithm 3 summarizes the SPEA2 procedure used in this study.
**Algorithm 3:** SPEA2 for ARMInput: Transaction set T, parameter set Output: Pareto-optimal rule set R 1: FI ← Apriori(T, s_start) 2: P ← Seed_and_RandomInitialize(FI, n_pop) 3: 𝒜 ← ∅ 4: for g = 0 to n_gen − 1 do 5:   min_s ← DynamicSupportThreshold(g) 6:   Evaluate(P, min_s) 7:   U ← P ∪ 𝒜 8:   Compute strength values for all solutions in U 9:   Compute raw fitness values using dominance relations 10:    Compute density values using nearest-neighbor distances 11:    Select feasible non-dominated solutions for the archive 12:    if |𝒜| > A_max then 13:      Apply archive truncation based on density 14:    end if 15:    Generate offspring population Q using tournament selection, binary DE/crossover, mutation, and repair 16:    Evaluate(Q, min_s) 17:    P ← Q 18: end for 19: R ← ParetoFiltering(P ∪ 𝒜) 20: return R

#### 2.14.3. Role of the Comparison Algorithms

The use of MOEA/D and SPEA2 provides two complementary perspectives for evaluating NSGA-II/DE-ARM. MOEA/D tests whether decomposition-based scalar subproblem optimization can effectively explore the four-objective ARM search space. SPEA2 tests whether strength-based elitist selection and density-based archive truncation can preserve high-quality association rules. In contrast, NSGA-II/DE-ARM relies on non-dominated sorting, crowding distance, lift-weighted tournament selection, and adaptive operator selection. Therefore, the comparison evaluates whether the proposed NSGA-II-based hybrid structure provides better Pareto front coverage and rule diversity than decomposition-based and strength-based alternatives under the same ARM-specific representation and constraints.

## 3. Results

### 3.1. Dataset

In this study, a multidimensional panel dataset has been produced and used. The curated dataset is composed of macroeconomic and governance variables obtained from the World Bank Worldwide Governance Indicators “https://databank.worldbank.org/source/worldwide-governance-indicators” (accessed on 20 January 2026) and the World Bank Open Data portal “https://data.worldbank.org” (accessed on 20 January 2026) the Economist Intelligence Unit Democracy Index “https://www.eiu.com/n/global-themes/democracy-index/” (accessed on 20 January 2026), and defense expenditure data from the Stockholm International Peace Research Institute (SIPRI) Military Expenditure Database “https://www.sipri.org/databases/milex” (accessed on 20 January 2026). The benchmark Mushroom dataset was obtained from the UCI Machine Learning Repository “https://archive.ics.uci.edu/dataset/73/mushroom” (accessed on 20 January 2026) [28]. The curated dataset covers the period 2002–2023 and consists of 3432 observations and 14 variables. Table 1 summarizes the variables used.

**Table 1:** Variables included in the dataset.

Abbreviation	Feature Name	Description	Type	Number of Categories
CoC	Control of Corruption	Control of Corruption	Numerical	3
GE	Government Effectiveness	Government Effectiveness	Numerical	3
PS	Political Stability and Absence of Violence/Terrorism	Political Stability Level and Risk of Violence/Terrorism	Numerical	3
RQ	Regulatory Quality	Impact of Policies and Regulations on the Private Sector	Numerical	3
RL	Rule of Law	Rule of Law and Trust in the Justice System	Numerical	3
VA	Voice and Accountability	Individuals’ Freedom of Expression and Level of Political Participation	Numerical	3
DEM	Democracy Index	Country’s Level of Democratic Development	Numerical	4
GDP	Gross Domestic Product (Economic)	Total Gross Domestic Product	Numerical	3
GDPPC	GDP per Capita (Economic)	GDP per Capita	Numerical	3
PC	Military Spending Per Capita	Military Expenditure Per Capita (Also Used as Target Variable)	Numerical	3
SGS	Share of Government Spending	Share of Government Spending in Total (Also Used as Target Variable)	Numerical	3
SGDP	Military Spending Share of GDP (Economic)	Military Expenditures as a Percentage of GDP (Also Used as Target Variable)	Numerical	3
ECAT	Economic Category	Country’s Level of Economic Development	Categorical	5
RCAT	Regional Category	The Country’s Regional Classification	Categorical	3

Numerical variables (except DEM) were transformed into quantile-based categories (Low, Medium, High). The DEM variable, on the other hand, was divided into four categories (Authoritarian Regime, Hybrid Regime, Flawed Democracy, Full Democracy) in accordance with the EIU classification. As a result, 46 unique items and 4612 frequent itemsets (min_supp = 0.10) were obtained.

The choice of transforming continuous variables into three equal-frequency (quantile) categories is grounded in three considerations. First, quantile-based discretization is a standard preprocessing step for continuous attributes in the association rule mining literature, as it preserves balanced support distributions across categories and avoids the support fragmentation caused by fixed-width binning of skewed variables. Second, from an information-theoretic perspective, too few categories (q = 2) weaken the discriminative capacity of variables, while too many categories (q ≥ 5) inflate the item space, push support values below practical thresholds, and substantially reduce the number of frequent itemsets. Third, WGI indicators are defined on an approximately [−2.5, +2.5] range with roughly symmetric distributions; a tertile split naturally aligns with Low/Medium/High governance quality tiers. The impact of this design choice on rule quality is empirically validated through the discretization sensitivity analysis in Section 3.14.

To evaluate the generalizability of the algorithms, the UCI Mushroom dataset, which is widely used in the association rule mining literature, was used as a second benchmark. The benchmark dataset consists of 8124 observations and 22 categorical variables (the single-valued variable—veil-type—was removed). By converting all categories of each variable into the “Variable_Value” format, 118 unique items were obtained; this results in an item space approximately 2.6 times larger than that of the SIPRI dataset. With Apriori frequent itemset mining (min_supp = 0.10), 287,215 frequent itemsets were obtained. Table 2 presents the comparative profile of the two datasets.

**Table 2:** Comparative profile of the datasets.

Feature	SIPRI	Mushroom
Number of observations	3432	8124
Number of variables	14	22
Unique items	46	118
Binary vector length	92 bit	236 bit
Frequent itemsets (min_supp = 0.10)	4612	287,215
Data type	Numerical → Categorical	Entirely categorical
Domain	Governance/Macroeconomics	Biology (mushroom classification)

### 3.2. Parameter Settings

The parameter values used for the three evolutionary algorithms are reported in Table 3. To ensure reproducibility and a fair comparison among the three algorithms, the parameters were determined as follows:

(i) Common evolutionary parameters. The population size (n_pop = 100), number of generations (n_gen = 120), and mutation probability (p_mut = 0.01) were set to values that are widely adopted in the multi-objective evolutionary ARM literature [13,15,16] and are consistent with the original NSGA-II [8], MOEA/D [9], and SPEA2 [10] reference implementations. Identical values were used across all three algorithms to isolate the effect of the algorithmic structure rather than the parameter configuration. The convergence behavior of all three algorithms was verified empirically (see Section 3.8, Convergence Analysis), confirming that n_gen = 120 is sufficient for stabilization.
(ii) ARM-specific parameters. The maximum rule length (max_rule_len = 6) and maximum consequent length (3) were chosen to balance rule expressiveness and interpretability, in line with common practice in evolutionary ARM [13,16]. The sensitivity of the algorithm to max_rule_len was further examined through a dedicated experiment (Section 3.13, Table 18), which confirmed that the chosen value yields the highest hypervolume. The initial and final minimum support values (0.08 → 0.03) were determined empirically based on preliminary frequent-itemset analysis on the SIPRI dataset; the dynamic decay schedule between these two bounds is described in Section 2.
(iii) Differential evolution parameters (NSGA-II/DE-ARM). The scaling factor (F = 0.55) and the crossover rate (CR = 0.85) lie within the recommended ranges for binary differential evolution reported by Krishna and Ravi [16] (F ∈ [0.4, 0.9]; CR ∈ [0.7, 0.9]). The crossover probability (0.90) follows the standard genetic operator recommendation. For MOEA/D and SPEA2, F = 0.40 was used as it is the value reported in their respective reference implementations [9,10].
(iv) Adaptive operator selection (AOS) parameters. The initial DE probability (p_DE_0_ = 0.75), the EMA smoothing factor (α_EMA = 0.10), and the bounds on the DE probability (p_min = 0.40, p_max = 0.85) were determined through a grid search over five candidate configurations, each evaluated with five independent seeds, and the configuration that produced the highest mean hypervolume on a held-out validation subset of the SIPRI dataset was retained. This procedure is described in Section 2 and is consistent with the credit-assignment-based AOS framework of Li et al. [31] and Fialho et al. [32].
(v) Algorithm-specific parameters. The number of neighbors for MOEA/D (15) follows the recommendation of Zhang and Li [9] for problems with up to four objectives. The external Pareto archive capacity (200) was set to twice the population size, which is a common heuristic to retain a sufficiently rich elite set without inflating computational cost.
(vi) Statistical analysis parameters. The number of bootstrap replications (500) for rule stability evaluation and the number of independent runs (30) for statistical comparison are both standard choices in the empirical evolutionary computation literature, providing sufficient power for the paired tests described in Section 3.4.

All numerical values reported in Table 3 are kept fixed throughout the experimental study; deviations from these values, where introduced for sensitivity analysis (e.g., max_rule_len), are described explicitly in the corresponding subsections.

**Table 3:** Algorithm parameters.

Parameter	NSGA-II/DE-ARM	MOEA/D	SPEA2
Population size (n_pop)	100	100	100
Number of generations (n_gen)	120	120	120
Mutation probability	0.01	0.01	0.01
Initial minimum support	0.08	0.08	0.08
Final minimum support	0.03	0.03	0.03
Maximum rule length	6	6	6
Maximum consequent length	3	3	3
Crossover probability	0.90	—	—
Initial DE probability (p_DE_0_)	0.75	—	—
AOS smoothing (α_EMA)	0.10	—	—
AOS minimum DE probability (p_min)	0.40	—	—
AOS maximum DE probability (p_max)	0.85	—	—
Scaling factor (F)	0.55	0.40	0.40
Crossover rate (CR)	0.85	0.85	0.85
Number of neighbors	—	15	—
Bootstrap replications	500	500	500
Archive capacity	200	200	200

### 3.3. Performance Metrics

- Hypervolume (HV): The volume covered by the Pareto front with respect to the reference point. A fixed reference point of r=[0, 0, −0.69, −1] was used. This point lies below the worst observed values in the four-objective space. The reference point was kept fixed across all runs and algorithms for comparison. HV was interpreted together with metric-based results (lift, confidence, support, and NetConf). It was calculated using the Monte Carlo method over 100,000 samples (coefficient of variation = 0.34%).
- Mean Lift, Confidence, Support, and NetConf: Reported as the average over 30 independent runs.
- Rationale for including NetConf as the fourth objective: NetConf reveals the relational structure that the confidence metric alone cannot capture by normalizing the effect of antecedent frequency. The substantial variation in the relationship between NetConf and confidence across datasets supports this choice. Indeed, the Pearson correlation was calculated as *r* = 0.97 for SIPRI and *r* = 0.24 for Mushroom. This indicates that NetConf carries different information across different item-space densities. A detailed correlation analysis is provided in Table 4.

**Table 4:** NetConf–confidence correlation analysis (at the Pareto rule level, seed = 42).

Dataset	Algorithm	n	Pearson r	*p*	Spearman ρ	Spearman *p*
SIPRI	NSGA-II/DE-ARM	78 ^1^	0.968	<10^−47^	0.916	<10^−32^
SIPRI	MOEA/D	29	0.948	<10^−14^	0.691	<10^−4^
SIPRI	SPEA2	97	0.978	<10^−65^	0.857	<10^−28^
Mushroom	NSGA-II/DE-ARM	52	0.186	0.186	0.340	0.014
Mushroom	MOEA/D	21	0.651	0.001	0.716	<10^−3^
Mushroom	SPEA2	36	0.091	0.597	0.407	0.014

^1^ The correlation analysis was conducted on the unique rule set obtained from the union of the population and the external Pareto archive (*n* = 78). The 68 rules in Table 5, however, belong only to the non-dominated Pareto front in the final generation.

**Table 5:** NSGA-II/DE-ARM Pareto front statistics (68 rules, seed = 42).

Metric	Min	25%	Median	Mean	75%	Max	Std
Lift	2.771	3.790	4.134	4.457	4.908	7.150	0.996
Confidence	0.470	0.841	0.944	0.893	0.982	1.000	0.121
Support	0.055	0.124	0.145	0.155	0.192	0.307	0.055
NetConf	0.469	0.779	0.857	0.826	0.905	0.944	0.102

- **Statistical tests:** For each metric, the normality of the difference distribution was checked using the Shapiro–Wilk test. When normality was satisfied, the paired *t*-test was applied; when it was not, the Wilcoxon signed-rank test was used. The Holm–Bonferroni method [33] was used for multiple-comparison correction. Effect size was reported using Cohen’s *d* [34]. For comparisons against the Apriori baseline (Table 8 and Table 13), the one-sample Cohen’s *d* formula was used: *d* = ( $\bar{x}$ − μ_0_)/s; where $\bar{x}$ is the mean of the 30 runs, μ_0_ is the Apriori reference value, and *s* is the standard deviation of the 30 runs (*ddof* = 1). For paired algorithm comparisons (Table 12), the formula *d* = $\bar{d}$/s_6_ was used, where $\bar{d}$ is the mean of the run-wise differences and s_6_ is the standard deviation of these differences.

To evaluate the overall ranking differences among the three algorithms simultaneously, the Friedman test—a non-parametric omnibus test for three or more related groups—was applied to each metric. For metrics in which the Friedman test yielded a statistically significant result, Holm-corrected pairwise Wilcoxon signed-rank comparisons were subsequently performed (Table 9, Table 10, Table 14 and Table 15).

### 3.4. Experimental Protocol

Each algorithm was run with 30 different random seeds (seed = 0, 1, …, 29). In each run, the average metric values were recorded, and the variability across runs was reported. The HV reference point was kept fixed across all runs to ensure comparability.

### 3.5. Pareto Front Analysis

The NSGA-II/DE-ARM algorithm discovered 68 Pareto-optimal rules in a representative run (seed = 42).

**Figure 1:**
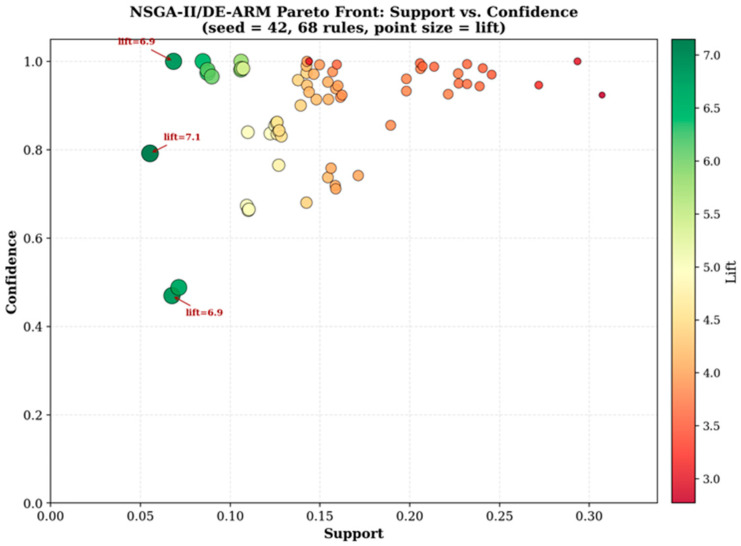
NSGA-II/DE-ARM Pareto front: support vs. confidence (point size = lift).

Figure 1 shows the distribution of the Pareto front in the support-confidence plane. The point sizes represent lift values.

Detailed statistics of the 68 Pareto-optimal rules generated by NSGA-II/DE-ARM are presented in Table 5.

#### 3.5.1. Rule Length Distribution

Table 6 shows the rule length distribution.

It is observed that rule length converges to the upper bound (max_rule_len = 6). This is because lift optimization rewards more specific rules. However, the presence of rules with lengths of 2 and 4 (10.3% in total) indicates that the Pareto front also preserves high-support compact rules.

#### 3.5.2. Thematic Analysis of Discovered Rules

Table 7 presents the 10 rules with the highest lift values on the Pareto front.

The examination of the discovered rules (68 rules) reveals four main thematic clusters among governance quality, economic structure, and defense expenditures.

**Theme 1.**  *Institutional Quality and the Defense Expenditure Paradox (Rules #1–10, lift > 5.797)*

*In the rules with the highest lift values, the following pattern is observed: PC_High (high military expenditure per capita) and SGDP_Low (a low share of military expenditure in GDP) appear together as antecedents, while SGS_Low (a low government spending share) emerges as the consequent (Rules #1, #2).*

*This association is consistent with the fact that developed economies make high defense expenditures in absolute terms (PC_High), while the proportional share of this expenditure remains low due to the size of their GDP (SGDP_Low).*

*These rules also include institutional clustering:*

- *CoC_High (control of corruption) and RL_High (rule of law) are observed together in many rules (Rules #2, #5, #8, #9, #10). This association is consistent with theoretical frameworks that emphasize the relationship between corruption control and legal assurance.*
- *VA_High (voice and accountability) appears in many high-lift rules (Rules #2, #3, #9, #10), indicating that democratic accountability occupies a central position in the institutional quality ecosystem.*

**Theme 2.**  *EU Convergence Pattern (Rules #11–25, lift 4.5–5.0):*

*Rules containing RCAT_EU and ECAT_HIGH are observed together with high GDPPC, GE, RL, and VA (Rules #15–25). The fact that the confidence values range between 0.70 and 0.86 indicates that these patterns may reflect the convergence observed between economic development and institutional quality in the EU region.*

**Theme 3.**  *Economic Structure–Governance Associations (Rules #28–52, lift 3.7–4.4):*

*Rules with moderate lift levels show that there are regular associations between macroeconomic indicators and governance quality. The frequent co-occurrence of the variables GDP_High, GDPPC_High, RQ_High, and GE_High suggests that the relationship between economic development and institutional quality is pronounced.*

**Theme 4.**  *Compact and High-Support Rules (Rules #61–65, length 2–4):*

*Short rules represent broad and strong association patterns:*

- *ECAT_HIGH → GDPPC_High (Rule #64, support = 0.293, confidence = 1.000): The strongest and most broad-based association in the dataset, indicating that all countries in the high economic category have high GDP per capita.*
- *RL_High → CoC_High (Rule #65, support = 0.307, confidence = 0.924): The relationship between rule of law and control of corruption is the highest-support rule in the dataset and holds in 31% of the observations.*
- *CoC_High, ECAT_HIGH → GDPPC_High, RL_High (Rule #61, support = 0.241, confidence = 0.985, length = 4): A compact rule showing that control of corruption and advanced economic status exhibit an almost deterministic association with high per capita income and legal assurance.*

This thematic analysis shows that the discovered association rules exhibit patterns consistent with the theoretical frameworks in the literature on institutional economics, development economics, and defense economics.

The confirmation of well-established macroeconomic relationships (e.g., the positive association between GDP and institutional quality) serves two methodological purposes. First, it validates the algorithm itself: when the evolutionary search consistently recovers relationships that are independently documented in the institutional economics literature [24,25,26], this is direct evidence that the four-objective optimization produces substantively meaningful rules rather than statistical artifacts. Second, several of the discovered patterns go further, revealing non-trivial conditional structures that conventional econometric analyses are not designed to capture. Three examples illustrate this. First, the defense expenditure paradox (Theme 1, Rules #1–10, lift > 5.8) shows that high per capita military spending (PC_High) co-occurs with a low GDP share of military expenditure (SGDP_Low) and a low government spending ratio (SGS_Low). The three-way pattern is well known at the aggregate level, but the rule places it within a single conditional structure together with institutional quality indicators (CoC_High, RL_High, VA_High), giving a more detailed reading of the same phenomenon. Second, the EU convergence pattern (Theme 2, Rules #11–25) captures multi-directional associations among regional membership (RCAT_EU), economic classification (ECAT_HIGH), and governance quality (GE, RL, VA) as a conditional co-occurrence structure—a form of relational knowledge that regression models, which test one dependent variable at a time, cannot express. Third, multi-consequent rules such as Rule #61 (CoC_High, ECAT_HIGH → GDPPC_High, RL_High) demonstrate near-deterministic joint outcomes (confidence = 0.985) that standard pairwise correlation analysis does not capture. The practical value of ARM in this setting lies in exactly this ability: expressing multi-directional, conditional co-occurrence patterns as interpretable decision-support rules. The three thematic examples above illustrate where this structural advantage over traditional econometric methods becomes visible.

From an algorithmic perspective, these results show that NSGA-II/DE-ARM can capture more complex association patterns that may be missed by the single-metric structure of Apriori.

### 3.6. 30-Run Statistical Benchmark

#### 3.6.1. NSGA-II/DE-ARM, MOEA/D and SPEA2 vs. Apriori (SIPRI)

Table 8 presents the statistical comparison of the 30 independent runs against the Apriori baseline. The normality assumption was checked using the Shapiro–Wilk test.

In cases where normality was not satisfied, the Wilcoxon signed-rank test was applied, whereas in cases where it was satisfied, the *t*-test was used. Multiple-comparison correction was performed using the Holm–Bonferroni method.

**Table 8:** 30-run statistical comparison (vs. Apriori).

Algorithm	Metric	Mean ± Std	95% CI	Apriori	Test	*p*-Value	Cohen’s d	Effect
NSGA-II/DE-ARM	Lift	4.748 ± 0.282	[4.643; 4.854]	3.646	Wilcoxon	<0.001	3.907	Large
NSGA-II/DE-ARM	Confidence	0.853 ± 0.034	[0.840; 0.866]	0.693	*t*-test	<0.001	4.711	Large
NSGA-II/DE-ARM	Support	0.146 ± 0.011	[0.141; 0.150]	0.125	Wilcoxon	<0.001	1.774	Large
NSGA-II/DE-ARM	NetConf	0.789 ± 0.031	[0.778; 0.800]	0.611	*t*-test	<0.001	5.766	Large
NSGA-II/DE-ARM	HV	3.231 ± 0.090	[3.197; 3.264]	0.000	*t*-test	<0.001	35.861	Large
MOEA/D	Lift	5.005 ± 0.352	[4.874; 5.137]	3.646	*t*-test	<0.001	3.859	Large
MOEA/D	Confidence	0.888 ± 0.037	[0.874; 0.902]	0.693	*t*-test	<0.001	5.240	Large
MOEA/D	Support	0.143 ± 0.012	[0.139; 0.148]	0.125	*t*-test	<0.001	1.466	Large
MOEA/D	NetConf	0.823 ± 0.034	[0.811; 0.836]	0.611	*t*-test	<0.001	6.274	Large
MOEA/D	HV	3.014 ± 0.122	[2.968; 3.059]	0.000	Wilcoxon	<0.001	24.745	Large
SPEA2	Lift	4.586 ± 0.148	[4.531; 4.641]	3.646	*t*-test	<0.001	6.337	Large
SPEA2	Confidence	0.879 ± 0.028	[0.868; 0.889]	0.693	*t*-test	<0.001	6.720	Large
SPEA2	Support	0.145 ± 0.009	[0.142; 0.148]	0.125	*t*-test	<0.001	2.272	Large
SPEA2	NetConf	0.807 ± 0.025	[0.798; 0.817]	0.611	*t*-test	<0.001	7.798	Large
SPEA2	HV	3.178 ± 0.076	[3.150; 3.207]	0.000	*t*-test	<0.001	41.799	Large

Significant after Holm–Bonferroni correction (α = 0.05). CI: Confidence Interval. HV reference: Since Apriori is a single-point solution, HV is approximately 0.

All three evolutionary algorithms demonstrate a statistically significant superiority over Apriori across all four metrics, with large effect sizes. In the hypervolume (HV) metric, NSGA-II/DE-ARM achieved the highest value (3.231 ± 0.090), showing a similar level to SPEA2 (3.178 ± 0.076), whereas MOEA/D (3.014 ± 0.122) exhibited lower HV.

#### 3.6.2. Friedman Test and Holm Post Hoc Analysis (SIPRI)

To assess the overall ranking differences among the three algorithms, the Friedman test was applied for each metric. The Friedman test is a non-parametric omnibus test that enables the simultaneous comparison of three or more related groups. For metrics in which the Friedman test was significant, Holm-corrected pairwise Wilcoxon signed-rank comparisons were performed.

**Table 9:** Friedman test results (SIPRI, 30 runs, 3 algorithms).

Metric	χ^2^	*p*-Value	Significant (α = 0.05)	NSGA-II/DE-ARM Rank	MOEA/D Rank	SPEA2 Rank
Lift	25.800	2.50 × 10^−6^	Yes	1.900	1.400	2.700
Confidence	7.400	0.025	Yes	2.367	1.967	1.667
Support	1.400	0.497	No	2.167	1.967	1.867
NetConf	6.467	0.039	Yes	2.367	1.733	1.900
HV	40.067	1.99 × 10^−9^	Yes	1.200	2.833	1.967

According to Table 9, the Friedman test results confirm that the ranking differences among the three algorithms are statistically significant in four of the five metrics (lift, confidence, NetConf, and HV). In the support metric, no significant difference was observed among the algorithms (χ^2^ = 1.400; *p* = 0.497).

In the HV metric, NSGA-II/DE-ARM holds a clearly superior mean rank (1.200), and the Holm post hoc analysis confirms that NSGA-II/DE-ARM is significantly superior to both MOEA/D (*p* = 4.26 × 10^−6^) and SPEA2 (*p* = 4.41 × 10^−5^). In the lift metric, MOEA/D holds the best rank (1.400) and shows a significant advantage over SPEA2, whereas the difference between NSGA-II/DE-ARM and MOEA/D remains above the significance threshold after Holm correction (*p* = 0.073). These findings are consistent with the pairwise tests reported below on Table 10.

**Table 10:** Holm post hoc pairwise comparisons (SIPRI, Friedman-significant metrics).

Metric	Comparison	Wilcoxon *p*	Holm-Adjusted *p*	Significant
Lift	MOEA/D vs. SPEA2	3.86 × 10^−7^	1.16 × 10^−6^	Yes
Lift	NSGA-II vs. SPEA2	7.06 × 10^−5^	1.41 × 10^−4^	Yes
Lift	NSGA-II vs. MOEA/D	0.073	0.073	No
Confidence	NSGA-II vs. SPEA2	0.005	0.016	Yes
Confidence	NSGA-II vs. MOEA/D	0.061	0.121	No
Confidence	MOEA/D vs. SPEA2	0.393	0.393	No
NetConf	NSGA-II vs. SPEA2	0.022	0.044	Yes
NetConf	NSGA-II vs. MOEA/D	0.019	0.056	No
HV	NSGA-II vs. MOEA/D	1.42 × 10^−6^	4.26 × 10^−6^	Yes
HV	MOEA/D vs. SPEA2	1.82 × 10^−5^	3.65 × 10^−5^	Yes
HV	NSGA-II vs. SPEA2	4.41 × 10^−5^	4.41 × 10^−5^	Yes

### 3.7. Ablation Study

The ablation study presented in Table 11 and the component contribution tests given in Table 12 reveal the following findings:

**Table 11:** Ablation study: 30-run averages (SIPRI dataset).

Variant	Lift ± Std	Confidence	Support	HV ± Std	Rule Count
LB + DE + CDP (Full)	4.671 ± 0.275	0.855	0.147	3.191 ± 0.097	80
DE + CDP	4.615 ± 0.266	0.864	0.149	3.197 ± 0.103	78
LB + CDP	4.668 ± 0.227	0.849	0.145	3.173 ± 0.080	76
LB + DE	4.565 ± 0.149	0.866	0.149	3.182 ± 0.084	78
CDP	4.595 ± 0.171	0.857	0.149	3.168 ± 0.063	72
LB	4.548 ± 0.169	0.856	0.150	3.169 ± 0.059	76
DE	4.521 ± 0.147	0.855	0.151	3.149 ± 0.076	76
Base	4.510 ± 0.143	0.869	0.152	3.176 ± 0.046	73

**Table 12:** Ablation paired comparison (30 runs, lift metric).

Comparison	Mean Difference	95% CI	*p*-Value	Cohen’s d	Effect
Full vs. Base	+0.161	[+0.041; +0.281]	0.010	0.503	Medium
CDP vs. Base	+0.086	[+0.004; +0.168]	0.041	0.391	Small
LB vs. Base	+0.038	[−0.039; +0.115]	0.316	0.186	Negligible
DE vs. Base	+0.012	[−0.066; +0.089]	0.757	0.057	Negligible
Full vs. LB + DE	+0.105	[−0.013; +0.223]	0.158	0.333	Small
Full vs. DE + CDP	+0.056	[−0.013; +0.125]	0.110	0.301	Small

Significant after Holm–Bonferroni correction (α = 0.05).

1. The full model (LB + DE + CDP) is significantly superior to the Base model (lift: *p* = 0.010; *d* = 0.503—medium effect; n_rules: *p* < 0.001; *d* = 0.891 large effect). The full model both increases lift and significantly expands the number of rules on the Pareto front.
2. Each component affects the metrics in different ways:
  - CDP: It is the strongest individual component in lift (+0.086; *p* = 0.041). It improves the quality of the Pareto front by eliminating infeasible solutions and tends to reduce the number of rules (72 vs. 73). This shows that it performs more selective filtering.
  - LB: It is not significant on its own in lift (*p* = 0.316). However, it reduces confidence (Base: 0.869 → LB: 0.856). This is evidence that LB preserves high-lift but low-confidence rules.
  - DE: It is not significant on its own in lift (*p* = 0.757). However, it increases the number of rules (73 → 76; *p* = 0.030). DE’s exploration capability produces more Pareto-optimal rules, but it does not increase their average lift on its own.
3. Synergy mechanism: The joint effect created by the LB and DE components is based on LB selectively preserving high-lift individuals from the broad candidate pool generated by DE. The decrease in confidence in the full model (0.869 → 0.855) and the increase in the number of rules (73 → 80) show that the Pareto front expands toward the low-confidence–high-lift direction.
4. HV metrics: The ablation test results (Table 11) show that the HV values vary between 3.149 and 3.197 across the variants. However, these differences do not reach the level of statistical significance.

This indicates that, although all variants produce a similar Pareto front volume, the full model shifts this volume toward the high-lift region.

The low variability of HV across variants (coefficient of variation < 3%) indicates that the fundamental Pareto structure of the four-objective optimization is preserved independently of the component. The HV convergence test confirms that the Monte Carlo estimation has a precision of coefficient of variation = 0.34%.

These findings show that the components do not significantly change the volume of the Pareto front (HV), but they can shift the position of the front toward the lift region.

### 3.8. Convergence Analysis

**Figure 2:**
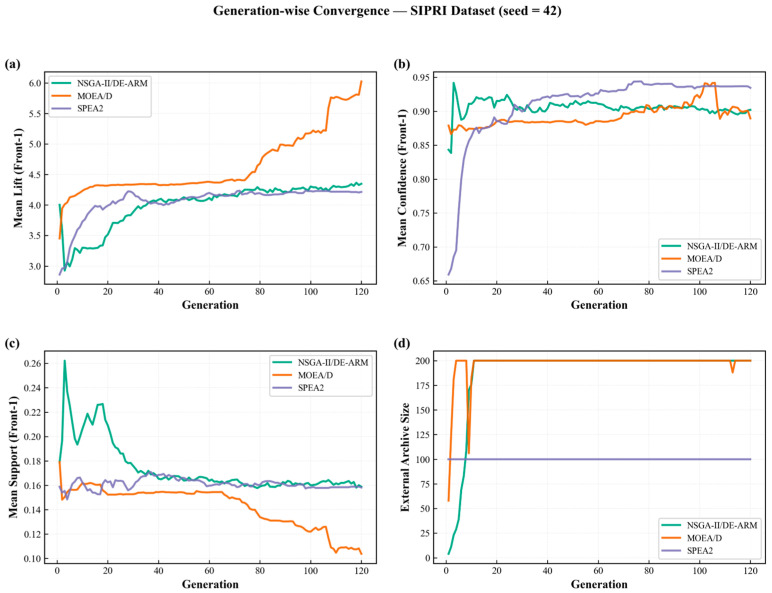
Generation-wise convergence: NSGA-II/DE-ARM (teal) vs. MOEA/D (orange) vs. SPEA2 (purple). (**a**) Mean Lift; (**b**) Mean Confidence; (**c**) Mean Support; (**d**) External Archive Size.

Figure 2 shows the generation-based convergence behavior of the three algorithms in four subplots. The monitored metrics are average lift, average confidence, average support, and archive size.

The following findings are obtained from the convergence plots:

1. Archive saturation: The archive size of NSGA-II/DE-ARM and MOEA/D reaches the maximum capacity (200) at approximately generations 15–20.
2. Metric stability: Average confidence and NetConf values begin to plateau after approximately generations 30–40. This finding shows that the algorithms reach convergence after this stage and that the remaining generations provide only limited contribution.
3. Lift convergence: The lift term reaches stability more slowly than the other metrics (around generation 60), reflecting that lift optimization is a more challenging task.
4. Difference between algorithms: MOEA/D appears to reach stability marginally faster in the confidence metric. SPEA2, on the other hand, reaches stability earlier due to its truncation-based environmental selection mechanism but remains at lower average lift values.

These convergence results show that the selected number of generations (*n_gen_* = 120) is sufficient for the convergence of all three algorithms.

### 3.9. Hypervolume Convergence Test

To verify the reliability of the Monte Carlo HV estimation, a convergence test was performed between 1000 and 500,000 samples. At the level of 100,000 samples, sufficient precision was achieved with a coefficient of variation of 0.34%; this value was used as the standard in all experiments.

### 3.10. Cross-Domain Validation: UCI Mushroom

To evaluate the generalizability of the algorithms, the same experimental protocol (30 independent runs, the same parameter set) was repeated on the UCI Mushroom dataset.

#### 3.10.1. NSGA-II/DE-ARM, MOEA/D and SPEA2 vs. Apriori (Mushroom)

Table 13 presents the statistical comparison of the 30 runs on the Mushroom dataset against the Apriori baseline.

**Table 13:** 30-run statistical comparison—Mushroom (vs. Apriori).

Algorithm	Metric	Mean ± Std	95% CI	Apriori	Test	*p*-Value	Cohen’s d	Effect
NSGA-II/DE-ARM	Lift	3.342 ± 1.871	[2.643; 4.040]	1.603	Wilcoxon	<0.001	0.929	Large
NSGA-II/DE-ARM	Confidence	0.904 ± 0.026	[0.895; 0.914]	0.744	Wilcoxon	<0.001	6.159	Large
NSGA-II/DE-ARM	Support	0.417 ± 0.030	[0.406; 0.428]	0.334	*t*-test	<0.001	2.749	Large
NSGA-II/DE-ARM	NetConf	0.686 ± 0.045	[0.669; 0.703]	0.473	*t*-test	<0.001	4.686	Large
NSGA-II/DE-ARM	HV	6.262 ± 0.724	[5.991; 6.532]	0.000	Wilcoxon	<0.001	8.650	Large
MOEA/D	Lift	4.537 ± 1.338	[4.038; 5.037]	1.603	Wilcoxon	<0.001	2.193	Large
MOEA/D	Confidence	0.961 ± 0.036	[0.947; 0.974]	0.744	Wilcoxon	<0.001	6.054	Large
MOEA/D	Support	0.253 ± 0.057	[0.232; 0.274]	0.334	*t*-test	<0.001	−1.416	Large (negative)
MOEA/D	NetConf	0.897 ± 0.073	[0.870; 0.924]	0.473	Wilcoxon	<0.001	5.791	Large
MOEA/D	HV	4.030 ± 1.102	[3.619; 4.442]	0.000	Wilcoxon	<0.001	3.656	Large
SPEA2	Lift	3.265 ± 0.592	[3.044; 3.486]	1.603	*t*-test	<0.001	2.809	Large
SPEA2	Confidence	0.925 ± 0.031	[0.913; 0.936]	0.744	*t*-test	<0.001	5.737	Large
SPEA2	Support	0.293 ± 0.035	[0.280; 0.306]	0.334	*t*-test	<0.001	−1.169	Large
SPEA2	NetConf	0.755 ± 0.092	[0.720; 0.789]	0.473	*t*-test	<0.001	3.058	Large
SPEA2	HV	4.415 ± 0.453	[4.246; 4.584]	0.000	*t*-test	<0.001	9.746	Large

Significant after Holm–Bonferroni correction (α = 0.05). CI: Confidence Interval. HV reference: Since Apriori is a single-point solution, HV is approximately 0.

As in the SIPRI dataset, all three evolutionary algorithms showed statistically significant improvements over Apriori in the lift and confidence metrics, with large effect sizes.

In the support metric, NSGA-II/DE-ARM significantly outperformed Apriori (*d* = 2.749). In contrast, MOEA/D remained below Apriori (*d* = −1.416), while SPEA2 showed a large decline (*d* = −1.169).

In the hypervolume (HV) comparison, NSGA-II/DE-ARM achieved the highest HV value (6.262 ± 0.724). This finding shows that the method has the broadest Pareto front coverage in the four-objective space.

This is because MOEA/D and SPEA2 focus on narrower, high-confidence subsets in Mushroom’s dense item space.

#### 3.10.2. Friedman Test and Holm Post Hoc Analysis (Mushroom)

The Friedman test was applied for the omnibus comparison on the Mushroom dataset, and Holm-corrected pairwise Wilcoxon signed-rank comparisons were performed for the significant metrics. The omnibus results are presented in Table 14, and the corresponding pairwise comparisons are reported in Table 15.

**Table 14:** Friedman test results (Mushroom, 30 runs, 3 algorithms).

Metric	χ^2^	*p*-Value	Significant (α = 0.05)	NSGA-II/DE-ARM Rank	MOEA/D Rank	SPEA2 Rank
Lift	32.267	9.85 × 10^−8^	Yes	2.733	1.267	2.000
Confidence	31.200	1.68 × 10^−7^	Yes	2.800	1.400	1.800
Support	50.867	9.00 × 10^−12^	Yes	1.033	2.867	2.100
NetConf	40.067	1.99 × 10^−9^	Yes	2.833	1.200	1.967
HV	54.200	1.70 × 10^−12^	Yes	1.033	2.933	2.033

**Table 15:** Holm post hoc pairwise comparisons (Mushroom, all metrics).

Metric	Comparison	Wilcoxon *p*	Holm-Adjusted *p*	Significant
Lift	MOEA/D vs. SPEA2	3.24 × 10^−6^	9.72 × 10^−6^	Yes
Lift	NSGA-II vs. MOEA/D	9.22 × 10^−6^	1.84 × 10^−5^	Yes
Lift	NSGA-II vs. SPEA2	0.002	0.002	Yes
Confidence	NSGA-II vs. MOEA/D	5.59 × 10^−9^	1.68 × 10^−8^	Yes
Confidence	NSGA-II vs. SPEA2	5.97 × 10^−6^	1.19 × 10^−5^	Yes
Confidence	MOEA/D vs. SPEA2	0.025	0.025	Yes
Support	NSGA-II vs. MOEA/D	1.86 × 10^−9^	5.59 × 10^−9^	Yes
Support	NSGA-II vs. SPEA2	5.59 × 10^−9^	1.12 × 10^−8^	Yes
Support	MOEA/D vs. SPEA2	4.41 × 10^−5^	4.41 × 10^−5^	Yes
NetConf	NSGA-II vs. MOEA/D	1.86 × 10^−9^	5.59 × 10^−9^	Yes
NetConf	MOEA/D vs. SPEA2	1.60 × 10^−5^	3.20 × 10^−5^	Yes
NetConf	NSGA-II vs. SPEA2	3.90 × 10^−5^	3.90 × 10^−5^	Yes
HV	NSGA-II vs. MOEA/D	1.86 × 10^−9^	5.59 × 10^−9^	Yes

In the Mushroom dataset, unlike SIPRI, the Friedman test detected highly significant ranking differences in all five metrics (all *p* < 10^−7^). The Holm post hoc analysis shows that all pairwise comparisons are also significant.

A particularly notable finding is that NSGA-II/DE-ARM achieves the best algorithm rank in nearly all runs for both the support metric (rank = 1.033) and the HV metric (rank = 1.033). MOEA/D, on the other hand, holds the best ranks in lift (1.267), confidence (1.400), and NetConf (1.200). This trade-off structure—NSGA-II/DE-ARM excelling in Pareto diversity and coverage while MOEA/D excels in individual metric optimization—directly supports the cross-dataset performance profile presented in Table 16 and the discussion in Section 4.

### 3.11. Cross-Dataset Performance Profile

A summary of the performance profiles in the two datasets is presented in Table 16.

The obtained test results reveal the following findings:

- The consistent superiority of NSGA-II/DE-ARM in the HV metric is preserved in both datasets (SIPRI: *d* = +1.35; Mushroom: d = +1.34 vs. MOEA/D). This finding confirms that the proposed framework’s capacity to produce the broadest Pareto front coverage in the four-objective space is domain-independent.
- MOEA/D’s consistent superiority in the confidence metric is preserved in both datasets (SIPRI: *d* = −0.61; Mushroom: *d* = −1.20).
- Differentiated performance in the lift metric: In SIPRI, NSGA-II/DE-ARM outperformed SPEA2 in lift (*d* = +0.54) and remained comparable to MOEA/D. In Mushroom, however, MOEA/D outperformed both NSGA-II/DE-ARM (*d* = −0.50) and SPEA2 (*d* = +0.92) in lift.

This indicates that the size of the item space can alter algorithm performance.

**Figure 3:**
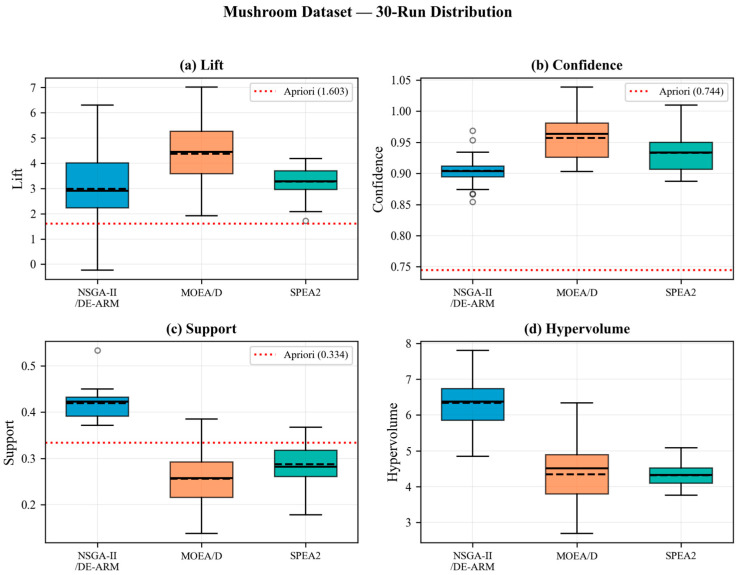
30-run distribution (Mushroom): NSGA-II/DE-ARM, MOEA/D, and SPEA2 vs. Apriori reference (red dashed line). (**a**) Lift; (**b**) Confidence; (**c**) Support; (**d**) Hypervolume.

- NSGA-II/DE-ARM’s marked superiority in the support metric is particularly notable in Mushroom (*d* = +2.50 vs. MOEA/D; *d* = +2.74 vs. SPEA2—large effect). The proposed framework appears to be effective in preserving high-support rules in large item spaces (118 items).
- All evolutionary algorithms achieved statistically significant improvement over Apriori in both datasets (*p* < 0.001), confirming the domain-independent generalizability of the many-objective evolutionary framework. Figure 3 shows the 30-run distribution in the Mushroom dataset.

### 3.12. Computational Cost

All experiments were conducted in Python 3.11 on an Intel Core i7-11800H (8 cores, 16 threads), 16 GB RAM, and Windows 11. The 30 independent runs were carried out using 8 parallel workers with ProcessPoolExecutor. Table 17 summarizes the computation times for each algorithm.

The higher computational cost of NSGA-II/DE-ARM compared to MOEA/D and SPEA2 arises from the runtime effect of two additional components: (i) the differential evolution (DE) operator requires three parent vectors in the mutation step and introduces additional crossover cost; (ii) adaptive operator selection (AOS) requires tracking operator success rates and updating probabilities in each generation.

In contrast, the total runtime remains at the level of minutes for both datasets and does not constitute a bottleneck in practical use. Most of the computational cost is dominated by the calculation of support, confidence, lift, and NetConf values for each individual, as well as bootstrap sampling of the stability metric (500 repetitions). This cost is common to all algorithms.

### 3.13. Rule Length Sensitivity Analysis

To evaluate the sensitivity of the proposed NSGA-II/DE-ARM algorithm to the maximum rule length (max_rule_len) parameter, five independent runs were conducted on the World Bank, EIU, and SIPRI dataset for max_rule_len ∈ {3, 4, 5, 6}. All other parameters were kept the same as in Table 3. Table 18 presents the mean and standard deviation values for each rule length constraint.

**Table 18:** NSGA-II/DE-ARM performance by maximum rule length (5-run mean ± σ; exploratory analysis).

max_rule_len	Rule	Lift	Confidence	Support	NetConf	HV
3	8.2 ± 4.2	3.196 ± 0.115	0.847 ± 0.121	0.219 ± 0.043	0.782 ± 0.114	2.318 ± 0.023
4	32.0 ± 9.2	3.857 ± 0.159	0.877 ± 0.024	0.166 ± 0.009	0.790 ± 0.014	2.838 ± 0.070
5	64.4 ± 8.3	4.270 ± 0.226	0.867 ± 0.033	0.158 ± 0.011	0.798 ± 0.025	3.042 ± 0.030
6	79.8 ± 7.5	4.545 ± 0.124	0.872 ± 0.034	0.153 ± 0.006	0.806 ± 0.028	3.172 ± 0.103

The results show that as the maximum rule length increases, the number of rules, the lift value, and the Pareto volume (HV) increase steadily. Support, on the other hand, decreases in the opposite direction. This trend shows that longer rules can reveal less frequent but more specific and more interesting association patterns. Confidence and NetConf values remained high and stable across all rule lengths (Confidence > 0.84; NetConf > 0.78). The obtained results show that the max_rule_len = 6 setting establishes a balance between lift and support and raises the Pareto volume to the highest level.

### 3.14. Discretization Sensitivity Analysis

To evaluate the impact of the number of quantile categories (q) on rule quality, a systematic sensitivity analysis was conducted on the WGI dataset for q = {2, 3, 4, 5}. In each configuration, DEM and categorical variables (ECAT, RCAT) were held constant; only the quantile count for numerical variables was varied. Five independent runs (seed = 42–46) were performed for each q value, with all other parameters identical to Table 3. Table 19 presents the item space profile and mean performance metrics for each discretization configuration.

Table 19 reveals the systematic impact of discretization granularity across five key dimensions: (1) Item space expansion—increasing q from 2 to 5 raises unique items from 35 to 68 (~1.9×) and binary vector length from 70 to 136 bits, causing exponential growth in the evolutionary search space. (2) Frequent itemset count—finer categories fragment support values, causing the FIS count to drop from 15,362 at q = 2 to only 564 at q = 5 (~27× reduction), directly affecting seed initialization quality. (3) Support-lift trade-off—as q increases, mean lift rises monotonically (3.26 → 8.78) while mean support decreases monotonically (0.210 → 0.075); at q = 5, support approaches the minimum support threshold (0.03–0.08), raising concerns about rule reliability. (4) Stability—rule count standard deviation increases substantially at q ≥ 4 (s = 3.3 at q = 3 vs. s = 20.5 at q = 5), indicating that finer discretization adversely affects evolutionary search stability. (5) HV—values are highest at q = 2 and q = 3 (3.260 and 3.228), with a slight decline at q = 4 (3.168) and high variance at q = 5 (3.209 ± 0.183).

In summary, the q = 3 configuration offers the most balanced profile in terms of support-lift trade-off, search space manageability, rule count stability, and HV performance. While q = 2 yields higher support, its low lift limits the capacity to capture meaningful association patterns. Configurations with q ≥ 4, despite exhibiting higher lift, suffer from support fragmentation, high variance, and a reduced number of frequent itemsets, which adversely affect practical applicability. These findings empirically validate that three-quantile discretization constitutes an optimal trade-off point in the ARM context.

## 4. Discussion

The experimental results reveal that evolutionary many-objective approaches provide more balanced and effective solutions compared to classical ARM methods. In both datasets (SIPRI and Mushroom), all three evolutionary algorithms achieved statistically significant improvements in lift and confidence metrics compared to Apriori (*p* < 0.001). This finding shows that the proposed many-objective evolutionary framework is applicable to different data domains in ARM problems.

In the SIPRI dataset, the three evolutionary algorithms exhibit different advantages. In the lift metric, MOEA/D demonstrated a moderate effect size advantage over NSGA-II/DE-ARM (d = −0.67). NSGA-II/DE-ARM showed a moderate advantage over SPEA2 in lift (d = +0.54). In the confidence metric, MOEA/D (d = −0.61) and SPEA2 (d = −0.59) showed an advantage over NSGA-II/DE-ARM with a moderate effect size.

In the Mushroom dataset, a different trade-off pattern emerged. In Mushroom, NSGA-II/DE-ARM lagged significantly behind MOEA/D in the lift metric (*p* = 0.005; d = −0.495); however, no significant difference was found between it and SPEA2 (*p* = 0.191). This situation stems from the high standard deviation of lift in NSGA-II/DE-ARM (1.871).

In contrast, NSGA-II/DE-ARM outperformed all algorithms in the support metric with a large effect size (d = +2.50 vs. MOEA/D; d = +2.74 vs. SPEA2).

The proposed framework appears to be effective in preserving high-support rules in large item spaces (118 items). The hypervolume (HV) comparison shows that NSGA-II/DE-ARM reached the highest HV value in both datasets (SIPRI: 3.231; Mushroom: 6.262). This consistency confirms that the proposed framework’s capacity to produce the broadest Pareto front coverage in the four-objective space is domain-independent.

A high HV means that the algorithm produces a broader and more diverse Pareto front in the four-objective space—this provides a richer set of options for decision-makers with different priorities. In the context of ARM, this is particularly important: an analyst may want precise but fewer rules (high confidence—an area where MOEA/D is strong), or may instead want to discover surprising yet statistically significant relationships (high lift, low confidence—a region included in the Pareto front of NSGA-II/DE-ARM). Indeed, among the 78 rules on the SIPRI Pareto front, 32 rules (41%) satisfying the condition confidence ≥ 0.90 provide confidence at the level of MOEA/D, while the remaining 46 rules include exploratory rules that MOEA/D could not reach. Single-metric optimization causes this exploratory space to be lost; the value of the many-objective Pareto approach stems precisely from this.

The ablation study (Table 11 and Table 12) provides empirical support for the component-level design rationale presented in Section 2. The full model (LB + DE + CDP; lift = 4.671) achieved a statistically significant improvement over the Base model (lift = 4.510) in terms of lift (Δ = +0.161; *p* = 0.010; d = 0.503), suggesting that the proposed components contribute more effectively when used jointly. Among the individual components, CDP provided the largest single improvement in lift (CDP: lift = 4.595 vs. Base: lift = 4.510; *p* = 0.041; d = 0.391), which is consistent with its intended role of maintaining feasibility pressure and reducing the tendency of the search process to drift toward high-lift but low-support regions. Lift-weighted selection (LB: lift = 4.548) and the DE operator (DE: lift = 4.521) also showed positive changes, although these improvements were not statistically significant when considered individually (*p* = 0.316 and *p* = 0.757, respectively). Overall, this pattern indicates that the components are functionally complementary: while some components provide limited gains in isolation, their combined deployment yields a clearer improvement over the Base model.

The discovered rules reveal meaningful associations between governance quality and economic indicators. Some of these associations, such as the positive relationship between GDP and institutional quality, are already well documented in the development economics literature. Therefore, the practical contribution of the ARM framework lies not in claiming novelty for individual pairwise relationships, but in identifying multi-variable and conditional co-occurrence structures that are difficult to summarize through simple pairwise analyses. For instance, the defense expenditure paradox (Theme 1) captures a specific interaction among absolute military spending, proportional military burden, and institutional quality within a single conditional rule. Such a pattern would typically require multiple model specifications or interaction terms in conventional econometric analysis, whereas ARM expresses it as a compact and interpretable rule. Furthermore, the multi-consequent rules, such as Rule #61 with confidence = 0.985, indicate strong joint outcomes across governance and economic dimensions, providing decision-makers with conditional patterns that complement marginal effect estimates. The consistency of these patterns with established theoretical frameworks [24,25,26] supports the substantive plausibility of the discovered rules, while their conditional specificity provides additional practical insight beyond simple pairwise associations.

The transformation of continuous variables into three quantile-based levels is a design choice, and different levels of granularity may directly affect rule quality. In this study, the three-level quantile discretization is justified by both common ARM practices [2,3] and the distributional characteristics of the WGI variables. The sensitivity analysis in Section 3.14 (Table 19) systematically compares q = {2, 3, 4, 5} configurations and shows that q = 3 provides the most balanced profile in terms of the support–lift trade-off, rule stability, and HV performance. Nevertheless, adapting the discretization level according to domain-specific granularity requirements remains a relevant direction for future work.

To enable a controlled comparison, MOEA/D and SPEA2 were implemented within the same infrastructure as NSGA-II/DE-ARM, using the same binary encoding, repair operator, and DE-based variation operators. This shared-infrastructure design was adopted deliberately to isolate the effects of selection and survival mechanisms (Section 2.14), in line with standard MOEA benchmarking practice [7,8]. However, this controlled setup does not evaluate the proposed framework against fully independent ARM-specific evolutionary methods, such as QuantMiner, G3P-based approaches [15], PSO-based ARM methods, WOA-based ARM methods, or recent nutcracker optimization variants [22], which employ different objective sets and encoding schemes. The next step is to adapt these methods to the four-objective setting used here and compare them more broadly. Nevertheless, the inclusion of the classical Apriori baseline with standard support–confidence filtering (Table 8 and Table 13) provides an independent non-evolutionary reference and supports the empirical advantage of evolutionary multi-objective optimization over conventional threshold-based ARM approaches in the present experimental setting.

The experiments were conducted on two datasets (SIPRI: 14 variables, 3432 observations; Mushroom: 22 variables, 8124 observations). In this way, the performance profiles of the algorithms were compared across different dimensionalities and domain contexts (social sciences vs. biology). However, validation is still required in different domains such as healthcare, finance, and market basket analysis, as well as on higher-dimensional datasets. In addition, increasing the number of objectives (for example, Certainty Factor or user-defined interestingness measures) may contribute to enriching the Pareto front.

## 5. Conclusions

In association rule mining, finding rules that simultaneously satisfy all quality criteria such as support, confidence, lift, and NetConf is a challenging problem. Because these criteria often exhibit conflicting tendencies with one another. NSGA-II/DE-ARM, developed in response to this need, adds components such as binary differential evolution operators, the constraint domination principle, an external Pareto archive, lift-weighted selection, and adaptive operator selection to the classical NSGA-II framework; thus, it aims to enhance both rule quality and solution diversity.

NSGA-II/DE-ARM presents a balanced multi-objective optimization framework for association rule mining. The main contribution of the method is that, rather than targeting consistent superiority in a single metric, it simultaneously optimizes four quality dimensions and offers the decision-maker a broad set of rules containing different solution balances.

Experiments conducted on two separate datasets, namely SIPRI and Mushroom, show that all three evolutionary algorithms achieved improvements in all metrics compared to Apriori. In comparisons among the evolutionary algorithms, it is observed that different advantages emerge in different metrics. MOEA/D and SPEA2 stand out in terms of average confidence and lift. NSGA-II/DE-ARM, on the other hand, produced the broadest Pareto front in both datasets in terms of the support metric and hypervolume (HV) analysis. This finding shows that the proposed method provides a more balanced and broader solution set in the four-objective space.

The significance of this study spans three complementary dimensions. From a methodological standpoint, the study extends multi-objective evolutionary ARM beyond the conventional two- or three-objective setting by jointly optimizing support, confidence, lift, and NetConf within a single binary-encoded search loop. Recent studies have investigated four-objective ARM formulations with different metric combinations [22,23]. However, the joint optimization of lift and NetConf together with constraint-domination, dynamic support thresholds, and adaptive operator selection has, to the best of the authors’ knowledge, received limited attention in evolutionary ARM. From an empirical standpoint, NSGA-II/DE-ARM generated broader Pareto fronts on both the SIPRI–World Bank and Mushroom datasets and outperformed the Apriori baseline with large effect sizes across all four metrics. From a practical standpoint, this study represents, to the best of the authors’ knowledge, an early application of many-objective evolutionary ARM to defense industry economics, a domain that remains largely unexplored in multi-criteria conditional pattern discovery. The conditional and multi-directional rules generated by the proposed framework provide a decision-support output that can complement conventional statistical analyses. The framework may also be transferable to other domains in which multi-criteria conditional patterns need to be extracted from categorical or discretized data, such as healthcare, economics, and the social sciences.

In the future, it is planned to incorporate rule comprehensibility into the model as an additional objective function, to make comparisons with other biologically inspired metaheuristic approaches such as ant colony and particle swarm, and to perform validation on datasets of different dimensions.

## Figures and Tables

**Table 6:** NSGA-II/DE-ARM rule length distribution (68 rules).

Rule Length	Count	Percentage
2	2	2.9%
4	5	7.4%
5	12	17.6%
6	49	72.1%

**Table 7:** Top 10 rules with the highest lift from the Pareto front.

#	Antecedent	Consequent	Support	Confidence	Lift	NetConf
1	GDPPC_High, PC_High, SGDP_Low	GDP_High, GE_High, SGS_Low	0.055	0.792	7.150	0.732
2	CoC_High, ECAT_HIGH, SGS_Low	PC_High, SGDP_Low, VA_High	0.068	0.470	6.859	0.469
3	PC_High, SGDP_Low, VA_High	ECAT_HIGH, PS_High, SGS_Low	0.068	1.000	6.850	0.917
4	ECAT_HIGH, RL_High, SGS_Low	GDPPC_High, RCAT_EU, SGDP_Low	0.071	0.488	6.673	0.486
5	CoC_High, ECAT_HIGH, SGDP_Low	GDPPC_High, RL_High, SGS_Low	0.085	1.000	6.463	0.924
6	ECAT_HIGH, RQ_High, SGDP_Low	GDPPC_High, PS_High, SGS_Low	0.087	0.974	6.284	0.900
7	ECAT_HIGH, PS_High, SGDP_Low	GDPPC_High, RQ_High, SGS_Low	0.087	0.980	6.084	0.899
8	CoC_High, GDPPC_High, SGDP_Low	GE_High, PS_High, SGS_Low	0.090	0.966	6.036	0.888
9	CoC_High, SGDP_Low, VA_High	PS_High, RL_High, SGS_Low	0.106	0.981	5.970	0.916
10	CoC_High, PS_High, SGDP_Low, VA_High	RL_High, SGS_Low	0.106	1.000	5.797	0.926

**Table 16:** Cross-dataset performance profile summary.

Comparison	Metric	SIPRI	Mushroom
NSGA-II vs. MOEA/D	Lift	MOEA/D superior (d = −0.67)	MOEA/D superior (d = −0.50)
NSGA-II vs. MOEA/D	Confidence	MOEA/D superior (d = −0.61)	MOEA/D superior (d = −1.20)
NSGA-II vs. MOEA/D	Support	No difference (*p* = 0.384)	NSGA-II superior (d = +2.50)
NSGA-II vs. MOEA/D	HV	NSGA-II superior (d = +1.35)	NSGA-II superior (d = +1.34)
NSGA-II vs. SPEA2	Lift	NSGA-II superior (d = +0.54)	No difference (*p* = 0.191)
NSGA-II vs. SPEA2	Confidence	SPEA2 superior (d = −0.59)	SPEA2 superior (d = −0.44)
NSGA-II vs. SPEA2	HV	NSGA-II superior (d = +0.55)	NSGA-II superior (d = +1.87)
MOEA/D vs. SPEA2	Lift	MOEA/D superior (d = +1.39)	MOEA/D superior (d = +0.53)
MOEA/D vs. SPEA2	HV	SPEA2 superior (d = −1.24)	No difference (*p* = 0.054)

**Table 17:** Computation times by algorithm (seconds).

Algorithm	SIPRI (Single Seed)	SIPRI (30 Seed)	Mushroom (Single Seed)	Mushroom (30 Seed)
NSGA-II/DE-ARM	~8	~38	~16	~468
MOEA/D	~5	~22	~7	~219
SPEA2	~6	~25	~10	~286

**Table 19:** Discretization sensitivity analysis by quantile count (WGI, 5-run mean ± s).

q	#Items	Binary Vec.	#FIS	#Rules	Lift	Conf.	Support	NetConf	HV
2	35	70 bits	15,362	84.4 ± 4.0	3.257 ± 0.134	0.864 ± 0.016	0.210 ± 0.009	0.744 ± 0.022	3.260 ± 0.087
3	46	92 bits	4612	72.8 ± 3.3	4.656 ± 0.193	0.874 ± 0.041	0.147 ± 0.010	0.807 ± 0.037	3.228 ± 0.068
4	57	114 bits	1525	66.2 ± 25.3	6.167 ± 0.467	0.854 ± 0.029	0.108 ± 0.016	0.804 ± 0.030	3.168 ± 0.065
5	68	136 bits	564	38.4 ± 20.5	8.776 ± 1.250	0.868 ± 0.014	0.075 ± 0.006	0.828 ± 0.020	3.209 ± 0.183

FIS: Frequent Itemset count (min_supp = 0.10).

## Data Availability

The original data presented in the study are openly available in “https://data.worldbank.org/” (accessed on 20 January 2026), “https://www.sipri.org/” (accessed on 20 January 2026), and “https://www.eiu.com/n/” (accessed on 20 January 2026).

## Abbreviations

The following abbreviations are used in this manuscript:

ARM	Association Rule Mining
NSGA-II	Non-dominated Sorting Genetic Algorithm II
MOEA/D	Multi-Objective Evolutionary Algorithm Based On Decomposition
SPEA2	Strength Pareto Evolutionary Algorithm 2
CDP	Constraint Domination Principle
AOS	Adaptive Operator Selection
HV	Hypervolume
DE	Differential Evolution
SIPRI	Stockholm International Peace Research Institute
ECAT	Economic Category
PC	Military Expenditure per Capita
SGDP	Military Expenditure as a Percentage of GDP
SGS	Government Spending Share
DEM	Democracy Index
CoC	Control of Corruption
GE	Government Effectiveness
PS	Political Stability
RQ	Regulatory Quality
RL	Rule of Law
VA	Voice and Accountability
GDP	Gross Domestic Product
GDPPC	GDP per Capita
RCAT	Regional Category
